# Impacts of the ‘Cost-of-Living Crisis’ on UK-Based Owners of Pandemic Puppies

**DOI:** 10.3390/ani16172656

**Published:** 2026-08-24

**Authors:** Claire L. Brand, Zoe Belshaw, Dan G. O’Neill, Gina T. Bryson, Rowena M. A. Packer

**Affiliations:** 1 Department of Clinical Science and Services, The Royal Veterinary College, Hawkshead Lane, North Mymms, Hatfield AL9 7TA, Herts, UK; clbrand@rvc.ac.uk (C.L.B.); gbryson19@rvc.ac.uk (G.T.B.); 2 EviVet Evidence-Based Veterinary Consultancy, Nottingham NG2 5HY, Nottinghamshire, UK; z.belshaw.97@cantab.net; 3 Department of Pathobiology and Population Sciences, The Royal Veterinary College, Hawkshead Lane, North Mymms, Hatfield AL9 7TA, Herts, UK; doneill@rvc.ac.uk

**Keywords:** dogs, welfare, health, behaviour, ownership, financial, Pandemic Puppies, veterinary

## Abstract

Since 2021, United Kingdom (UK) residents have faced a decline in disposable income termed the Cost-of-Living Crisis (COLC). For pet owners, this has been compounded by the costs of veterinary care rising faster than the rate of inflation. Animal welfare charities have reported increased rates of relinquishment and owners making cost-reducing savings on multiple aspects of their dogs’ care. This study aimed to quantitatively assess the nature and prevalence of impacts of the COLC on a cohort of dogs acquired in 2020 and their owners. A cross-sectional survey collected data between 2022 and 2024 from *n* = 770 owners of “Pandemic Puppies” as their dogs reached 36 months of age. Questions focused on the impacts of COLC on household income and expenditure, as well as on specific aspects of dog management. Results identified our owner cohort to have a modal household income which was three times higher than the average household income. Despite this, 36% reported that their household income had been negatively impacted by COLC. Over one in ten (12.4%) owners reported having made COLC-driven negative changes to their dogs’ care, with 10.8% anticipating making changes in the future. The most common changes were reduction in or cessation of pet insurance cover, reduced use of professional dog trainers, groomer and daycare services and delays to seeking veterinary attention for potential health problems. Given the relative affluence of our respondents and the specific features of this “Pandemic Puppy” cohort, these results may not be widely generalisable and may substantially underestimate the negative impacts of the COLC on canine welfare in the wider UK population.

## Simple Summary

Residents of the United Kingdom have recently experienced a rise in the cost of goods and services called “the Cost-of-Living Crisis”. This has led to increasing numbers of dog owners rehoming their dogs or trying to reduce their care costs. This study aimed to find out how the Cost-of-Living Crisis had affected a group of dogs bought as puppies during 2020. We collected survey data between 2022 and 2024 from 770 dog owners of “Pandemic Puppies” as their dogs reached 36 months of age. Despite 71.6% of these owners’ reporting household incomes exceeding the national average, approximately one third reported that their household income had reduced due to the Cost-of-Living Crisis. Over 10% had already made changes to their dogs’ care due to the Cost-of-Living Crisis, with 10% expecting to do so in the future. The most commonly reported changes were cancelling or reducing pet insurance cover, reducing the use of dog trainers, groomers and dog daycare, and delays in taking their dog to see a veterinary surgeon. These results suggest that owned-dog welfare in the United Kingdom has been impacted by the Cost-of-Living Crisis and that this impact is likely to be ongoing.

## 1. Introduction

Since 2021, United Kingdom (UK) households have faced a decline in disposable income as prices for essential goods and services have outpaced stagnant or slowly increasing wages [1]. Contributing factors to the ‘Cost-of-Living Crisis’ (COLC) include the COVID-19 pandemic, global supply chain disruptions, surging inflation, increased taxation, and a dramatic spike in energy prices influenced by international conflicts and economic instability [2]. UK households have consequently been forced to redefine what outgoings are necessary versus dispensable [3], including expenditure on pets [4].

Approximately one third of UK households own at least one dog [5], equating to around 13.5 million dogs [6] following a sharp increase in dog acquisitions during the COVID-19 pandemic [7,8]. Dogs in the UK are often regarded as family members, with their well-being interwoven with that of their owners [9,10]. However, dog ownership comes with responsibilities [11], including financial commitments for food, veterinary care, grooming, and other provisions deemed necessary by owners to optimise their dogs’ welfare [12]. The COLC has directly impacted the magnitude of these financial commitments [4,13]. By 2023, the cost of feeding a dog had risen by an estimated cumulative 32%, significantly outpacing cumulative general inflation (8%) [14], while veterinary costs increased by a cumulative 63% between 2016 and 2023 [15] and dog insurance premiums increased cumulatively by 1% between 2024 and 2025 [16]. As a result, between 2022 and 2023, an increasing number of pet owners (rising from 68% to 81%) reported that the costs of looking after their pets were higher, with 24% feeling that their pets had suffered as a result [17].

Since the COVID-19 pandemic, UK dog ownership demographics have shifted towards groups identified as more vulnerable to the financial responsibilities of dog ownership. These include first-time owners, owners of pets acquired during the COVID-19 pandemic, owners with children in their household and owners of dogs with behavioural problems [7,8,18,19,20]. Supporting a differential impact on young owners, the RSPCA report that owners aged 18–24 years were most concerned about affording the essential welfare needs of their pet (dogs and other companion animal species) [21]. This may reflect unrealistic expectations of pet care costs, with a UK Pet Food survey finding that almost one quarter (23%) of 16–24 year olds who owned a pet reported ownership as more expensive than they had expected, compared to 18% of the general population [20].

Animal charities including the RSPCA, PDSA and Dogs Trust have identified multiple specific animal welfare concerns related to the COLC [4,22,23,24,25]. These include more owners choosing not to register their pet with a veterinary practice [24]; delaying taking their ill pet to see a veterinarian [24]; being unable to pay for veterinary bills [26]; choosing not to have their pet neutered [24] or insured [24,27]; and considering giving human medicine to their pet to avoid the cost of veterinary ones [24]. Potentially compounding this, a recent report [4,28] highlights that deprived and urban locations in England and Wales are less likely to have accessible veterinary care, reducing those owners’ ability to find a veterinary practice which best matches their budget. In addition, pet owners report that discussions about costs with veterinary staff can be challenging and sometimes poorly transparent [29]. For some owners fortunate enough to live within their catchments, charity veterinary clinics such as the Blue Cross [4] have provided an invaluable low-cost service. Several UK dog rehoming organisations have also reported increasing demands on their services through the COLC, with increased relinquishment rates twinned with reductions in applications to adopt [24,30,31,32]. The numbers of stray dogs taken into Local Authority shelters in the UK and Ireland also increased between 2021 and 2023 [33]. Furthermore, RSPCA data over a four-year period from 2018 to 2022 indicate a reduction in the adoption of dogs during the COLC (defined as October 2021 onwards) compared to before [34].

The well-being of some UK-based pet owners has also been negatively affected by the COLC [4]. Owners are reported to have taken austerity measures when caring for themselves in order to prioritise their pets, including reducing spending on their weekly food shop and skipping meals, giving up personal luxuries, reducing household energy consumption and reducing/stopping charitable donations [24]. This echoes previous findings that when forced to choose, many owners will prioritise their pets’ needs at the expense of their own [35,36,37]. One third of owners (32%) in a national survey by Blue Cross stated that COLC pressures and the need to care for their pet had negatively impacted their mental health [26]; interviews conducted by Muldoon and Williams [4] with owners who had used Blue Cross services during the COLC lay bare the stress associated with being unable to afford a pet’s care, particularly in emergency situations. Nearly half (47%) of the owners in a PDSA report stated they were worried about affording the cost of treatment should their pet fall ill or be injured, with one quarter already having used credit cards or fallen into debt to afford unexpected veterinary treatment [24]. These cost of treatment impacts are likely to be particularly heightened for owners of animals with significant health or behavioural issues [4], both of which are common in the UK dog population [7,8,18].

Charity and industry reports have provided important evidence on the scale and impact of the COLC on UK pets and their owners. However, there remains a need for further empirical research to explore the impact of the economic crisis on canine and human welfare in the UK. Given ongoing concerns regarding the affordability of pet ownership, particularly in relation to veterinary bills and pet insurance [21], the relevance of the COLC to animal welfare remains high.

This study therefore aimed to quantitatively assess the nature and prevalence of impacts of the COLC on owners and their dogs acquired in 2020 during the COVID-19 pandemic (“Pandemic Puppies”) before the start of the COLC. As a result, no prior hypotheses were generated for this study. This work is part of an ongoing longitudinal study of the health, behaviour, and dog–owner relationship in this cohort [18].

## 2. Materials and Methods

### 2.1. Survey Design and Data Collection

A cross-sectional online survey was designed iteratively amongst the authors using novel questions to examine the impact of the COLC on owners and their dogs aged 36 months in the UK as part of the larger (“Pandemic Puppies”) longitudinal study [18]. Ethical approval for this study was granted by The Social Science Research Ethical Review Board at the Royal Veterinary College (RVC; URN: SR2020-0259). The COLC section of the survey was piloted upon a small number (*n* = 8) of respondents to assess readability and ease of completion.

**Figure 1:**
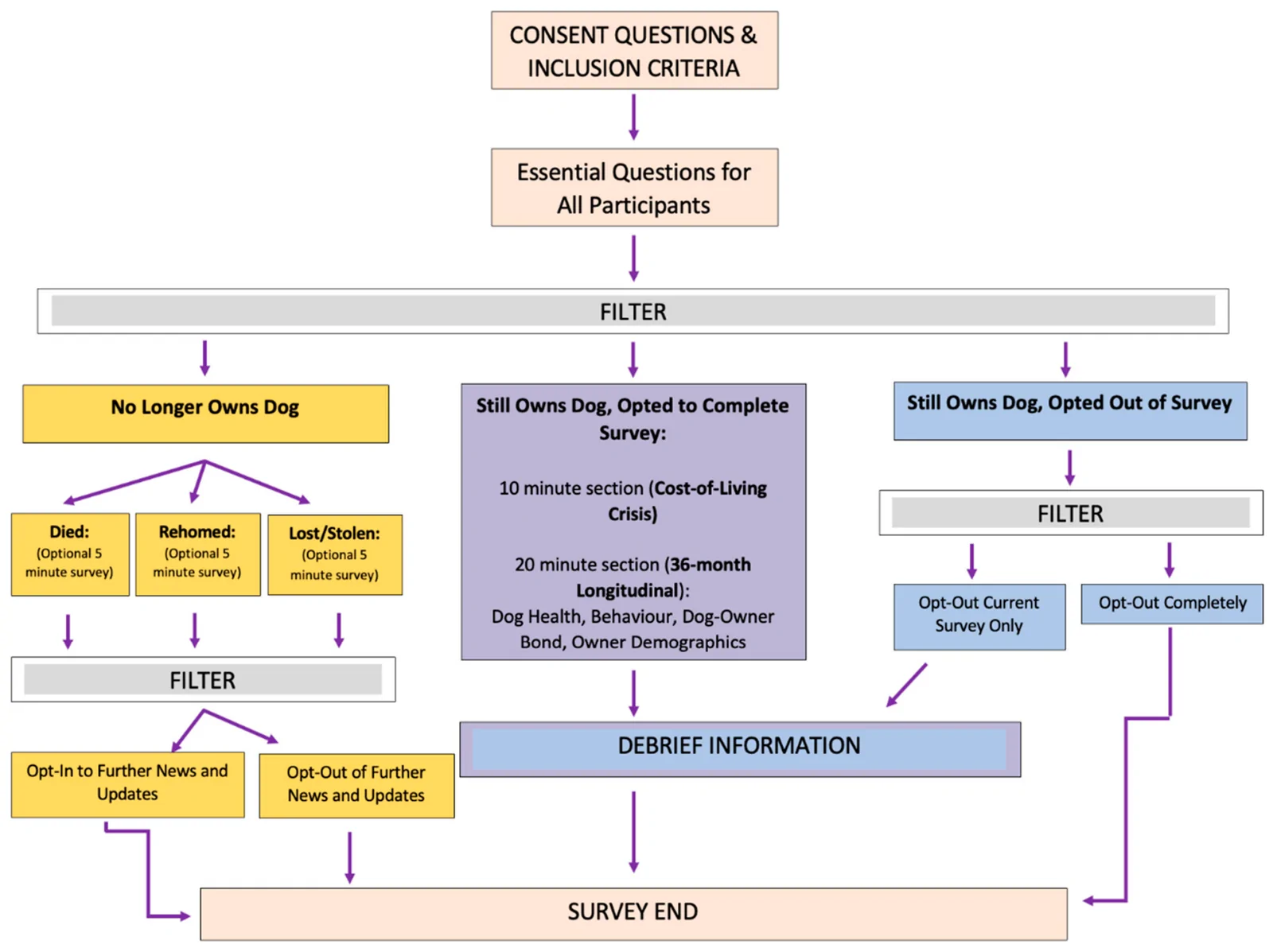
Schematic representation of the survey structure used for UK-based owners of 36-month-old dogs at the time of the survey (23 November 2022–4 January 2024) purchased under 16 weeks of age between 23 March and 31 December 2020. An option to opt out of the survey and/or all future contact was also provided. Respondents indicating they were happy to proceed were directed to the remainder of the survey. Following completion of the ‘Cost-of-Living Crisis’ (COLC) section, respondents were directed to the 36-month longitudinal section which formed part of a larger longitudinal study.

The survey was hosted by the RVC via the Vanderbilt University’s Research Electronic Data Capture platform (REDCap) [38,39]. Respondents to the original 2020 ‘Pandemic Puppies’ survey [7,8] who opted in to further research and provided a valid email address (*n* = 2366) were directly emailed a unique direct link to the COLC survey 14 days before their dog reached 36 months of age (commencing 23 November 2022 and ending 4 January 2024), with the link inactivated 28 days after sending. Reminder emails were sent to invited respondents who had not started or completed the survey at 14 and 26 days. All respondents were required to give informed consent prior to beginning the survey. All respondents, including those who no longer owned their dog, were able to opt out of future studies (Figure 1).

### 2.2. Survey Content

All survey questions relevant to the current study can be found in Supplementary File S1. The COLC section (Figure 1) examined impacts across six areas: (i) changes in ownership status, e.g., whether the dog was still owned by the respondent by 36 months of age and, in the case of relinquishment, whether the COLC had impacted upon this outcome; (ii) impacts upon canine health care decision-making, e.g., seeking advice from veterinary surgeons, use of preventative parasite control, vaccinations and insurance cover; (iii) impacts upon advice-seeking for dog behavioural issues and attendance at training classes; (iv) impacts upon canine daily maintenance, e.g., how often dogs were groomed, changes in paid daycare for dogs and changes in the dogs’ regular diet; (v) impacts upon household finances, respondents’ perception of impacts on dog welfare, and whether owning a dog had impacted financially upon respondents’ lifestyle; and (vi) the owner’s socioeconomic data, e.g., annual household income and highest level of education attained.

The remaining 36-month longitudinal section (Figure 1) additionally covered (vii) dog behaviour including owner-reported behaviour problems and separation-related behaviours (SRBs), (viii) dog health in terms of owner-reported transient and ongoing health disorders, (ix) owner expectations versus realities of dog ownership, and (x) owner demographics that could change over time (time-varying variables including UK region of residence, household composition, employment status). All questions in both sections were optional.

Canine demographics (e.g., breed, sex), other fixed owner demographics (e.g., prior dog ownership experience) and motivations/behaviours at the point of dog purchase were included from the original ‘2020 Pandemic Puppies’ survey [7,8]. Further details of the variables used in this study and from which survey they were obtained (‘2020’, ‘36-month longitudinal’ or ‘COLC’) are included in Table S1, Supplementary File S2.

### 2.3. Data Cleaning and Categorisation

Prior to analysis, raw data were imported into Microsoft Excel for Mac (version 16.79.2) for data cleaning. The status of time-varying respondent demographic variables that could have changed between the original ‘2020 Pandemic Puppies’ survey and the current survey were checked and updated as necessary. Respondents’ ages were not re-collected, so they are classified based on their reported age in 2020. Responses to multiple-choice questions (MCQs) of ‘I can’t remember’/‘I’d rather not say’/‘Prefer not to say’ and ‘Not applicable’ were categorised as missing data.

Respondents’ highest achieved UK-based educational qualifications were assigned to one of eight levels used in England, Wales and Northern Ireland [40]. The Scottish options of ‘Higher grade/advanced higher (Scotland)’ and ‘Certificate of sixth year studies’ were assigned to the same educational level as ‘A-Level’. The first half of participants’ postcodes were checked for validity against the Office for National Statistics (ONS) National Statistics Postcode Lookup (NSPL) May 2023 data [41] and allocated to one of the 12 UK regions.

Classification of dog breeds and dogs’ mean adult bodyweight was derived from VetCompass data for the breed as previously described [7]. Categorisation of owner-reported problem behaviours and separation-related behaviours was performed as described [18]. Further methodological details of these categorisations are included in Supplementary File S2.

Qualitative content analysis of free-text ‘other’ options to multiple-choice questions was applied as previously described [7,8,18]. All free-text responses for the COLC questions were deemed to fit within existing fixed-choice options and were therefore back-allocated into the relevant category.

#### Specific Impacts of COLC on Aspects of the Dogs’ Welfare

To summarise the potential impacts of COLC-influenced management changes made by owners on dogs’ welfare, four categories of impact were created: no change; potentially positive change for canine welfare; potentially negative change for canine welfare; and ambiguous change. This was done through consulting existing animal welfare codes, veterinary literature (see Table S2, Supplementary File S2), followed by discussion between the authors (a team of experienced animal welfare scientists and veterinary surgeons) to discuss the relative balance of benefits and harms. Where evidence was weak or conflicting, the authors used their cumulative professional experience to assign the category. This process was followed for each of the multiple-choice responses to 14 questions relating to changes made to: dog healthcare and behaviour advice seeking; dog training; diet; grooming; use of paid daycare services (dog walkers and doggy daycare); and use of boarding kennels for holidays (Table S2, Supplementary File S2). Decisions related to classification of breeding and neutering as “ambiguous” were based on potential impact to the welfare of individual dogs rather than the whole UK dog population. Any disagreements between the author team were resolved by discussion.

Responses to the Likert questions regarding whether the COLC had negatively impacted or were anticipated to negatively impact how respondents had managed/looked after their dog as well as whether owning a dog during the COLC had, or were anticipated to, affect respondents’ financial decisions regarding their own lifestyle were collapsed into binary outcomes. ‘Very much’, ‘Quite a lot’ and ‘A little bit’ were categorised as ‘1’, and ‘Not at all’ was categorised as ‘0’.

### 2.4. Statistical Analysis

Cleaned data were imported into IBM SPSS Statistics v29 (SPSS Inc., Chicago, IL., USA). Descriptive statistics (frequency and percentage) were calculated for all variables. The distribution of continuous data was ascertained through visual inspection of histograms, with results reported as mean ± standard deviation (SD) for normally distributed variables and median and range (inter-quartile range [IQR]) for non-normally distributed variables. Annual household income was compared to the national median UK average for 2023 [1] using chi-square (χ^2^) analysis. Risk factor analysis for binary categorical outcomes used univariable binary logistic regression, with 95% confidence interval (CI) and unadjusted odds ratio (uOR) reported. Statistical significance was set at 5%. Only univariable modelling was used due to the study being exploratory in nature with no prior hypothesis.

## 3. Results

A total of *n* = 775 valid responses were received from *n* = 2366 email invitations (32.76% response rate), of which *n* = 770 (99.35%) respondents still owned their dog. Of the five dogs no longer owned, three had died and two had been rehomed; COLC was not a factor in these outcomes.

### 3.1. Owner/Household Demographics

Most respondents were female (*n* = 695/769, 90.38%), and the most common age group of owners at the time of their puppy’s acquisition was 45–54 years (*n* = 215/769, 27.96%; Table S3, Supplementary File S2). A minority of owners lived alone or were the only adult in the household (*n* = 123/752, 16.36%), whilst just under one in four owners co-habited with a child ≤ 18 years of age (*n* = 148/632, 23.42%; Table S3, Supplementary File S2). Most respondents owned one dog (*n* = 505/768, 65.76%); *n* = 263/768 (34.24%) owned more than one dog and/or previously owned another dog (*n* = 522/769; 67.88%). Most respondents were employed (*n* = 476/712, 66.85%; 2023 UK national average employment rate: 75.70% [42]) and educated to level 4 or above (*n* = 512/731, 70.04%; 2023 UK national average: 48.40% [43]; Table S3, Supplementary File S2).

### 3.2. Dog Demographics

Most dogs whose breed status was provided were purebred (*n* = 550/769, 71.52%), whilst *n* = 190/769 (24.70%) were designer crossbreed, of which *n* = 140/190 (73.68%) were poodle-type crosses (Table S4, Supplementary File S2). The three most common breeds/crossbreeds were Labrador Retriever (*n* = 80/774, 10.34%), Cockapoo (*n* = 59/774, 7.62%) and Cocker Spaniel (*n* = 48/774, 6.20%). Most were neutered (*n* = 551/751, 73.37%), with sex similarly distributed (male *n* = 395/769, 51.37%; Table S4, Supplementary File S2). Over one third of dogs based on VetCompass typical breed adult bodyweight were categorised as weighing 10 to <20 kg (*n* = 282/747, 37.75%; Table S4, Supplementary File S2).

### 3.3. Respondents’ Annual Household Income and Geographic Location

**Figure 2:**
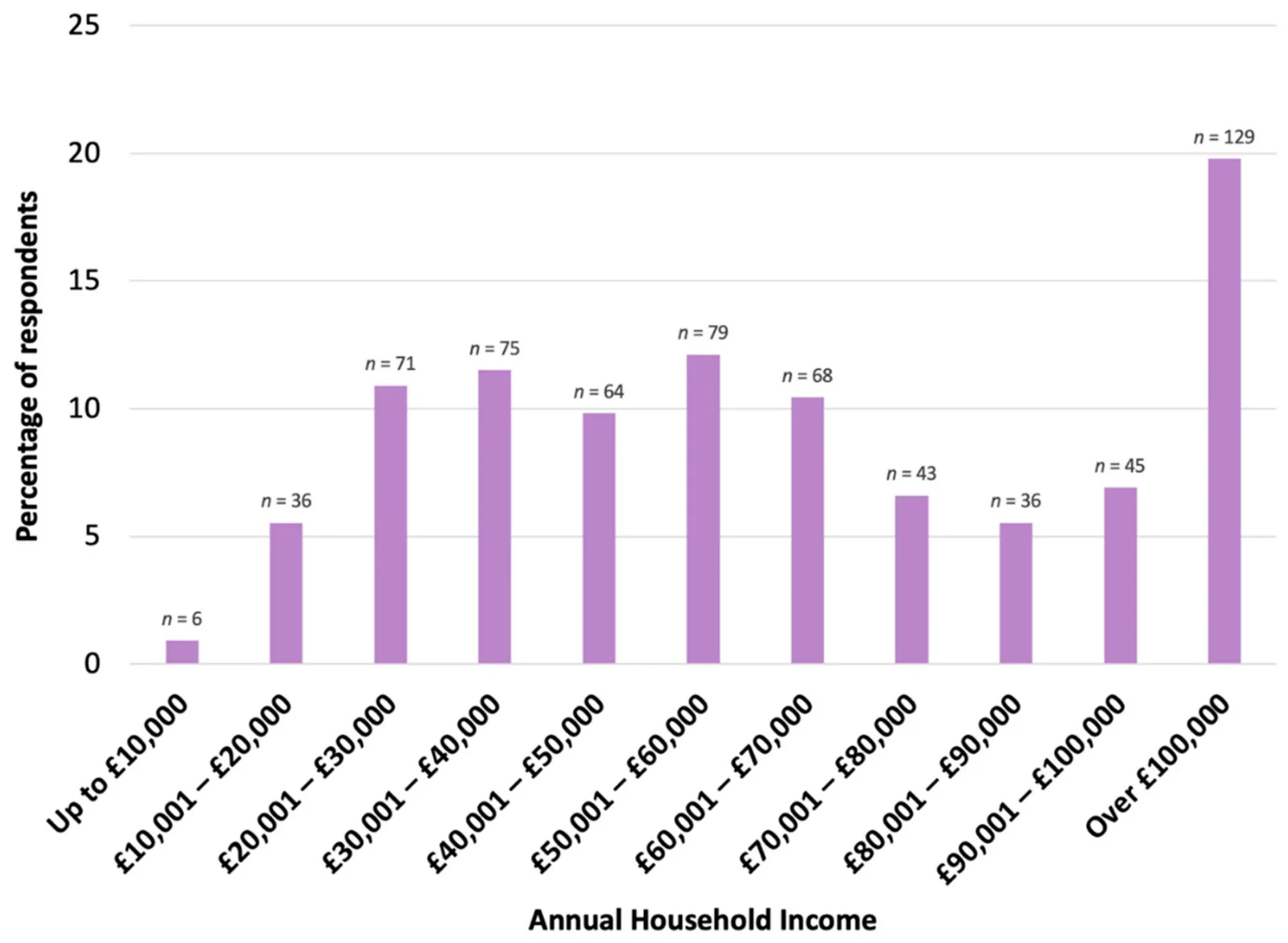
Responses to the question “What is your overall annual household income?” (*n* = 652) amongst UK-based owners of 36-month-old dogs purchased under 16 weeks of age between 23 March and 31 December 2020. The response option “Prefer not to say” was not included in the analysis (*n* = 102 responses). Total valid responses to the survey, *n* = 770.

The modal category of annual household income at the time of survey completion (23 November 2022–4 January 2024) was ≥ £100,001 (*n* = 129/652, 19.79%; Figure 2). The UK national median annual household income in 2023 was £34,500 [1]; this was exceeded by 71.62% of respondents. The UK regional distribution of respondents did not differ significantly from the mid-2023 UK distribution [44]; χ^2^ = 5.251, df = 11, *p* = 0.918.

### 3.4. Current and Anticipated Impacts of the COLC upon Household Finances, Dog Management and Household Lifestyle Decisions

A total of *n* = 381/760 (50.14%) respondents reported that their household finances had not changed in the preceding nine months, with *n* = 281/760 (36.97%) indicating that their household finances were worse, *n* = 79/760 (10.39%) that their household finances were better, and *n* = 6/760 (0.79%) unsure.

At least 25.58% of respondents when categorised by household income bracket reported that their household finances were worse than nine months previously (≤£30,000: *n* = 50/109, 45.87%; £30,001–£60,000: *n* = 89/216, 41.20%; £60,001–£90,000: *n* = 52/145, 42.76%; ≥£90,001: 44/172, 25.58%).

At least 36.16% of respondents in all age categories reported that their household finances were worse than nine months previously (18–24: *n* = 11/20, 55.00%; 25–34: *n* = 38/98, 38.78%; 35–44: *n* = 47/121, 38.84%; 45–54: *n* = 83/207, 40.10%; 55–64: *n* = 64/177, 36.16%; 65–74: *n* = 30/104, 71.15%; ≥75: *n* = 7/13, 53.85%). Respondents aged 25–34 and 35–44 years were the most likely to report that their finances had improved in the previous nine months compared to 45–54 year olds (25–34 years: *n* = 20/98, 20.41%, uOR 3.061, CI 1.51–6.22, *p* = 0.002; 35–44 years: *n* = 22/121, 18.18%, uOR 2.653, CI 1.33–5.28, *p* = 0.005).

Respondents whose household finances had improved or remained the same compared to the previous nine months were less likely to report negative impacts on the way they managed their dog compared to those who reported their household finances were worse than nine months previously (finances improved: *n* = 73/78, 93.60%, uOR 0.222, CI 0.09–0.57, *p* = 0.002; finances the same: *n* = 351/378, 92.86%, uOR 0.249, CI 0.16–0.40, *p* < 0.001).

**Figure 3:**
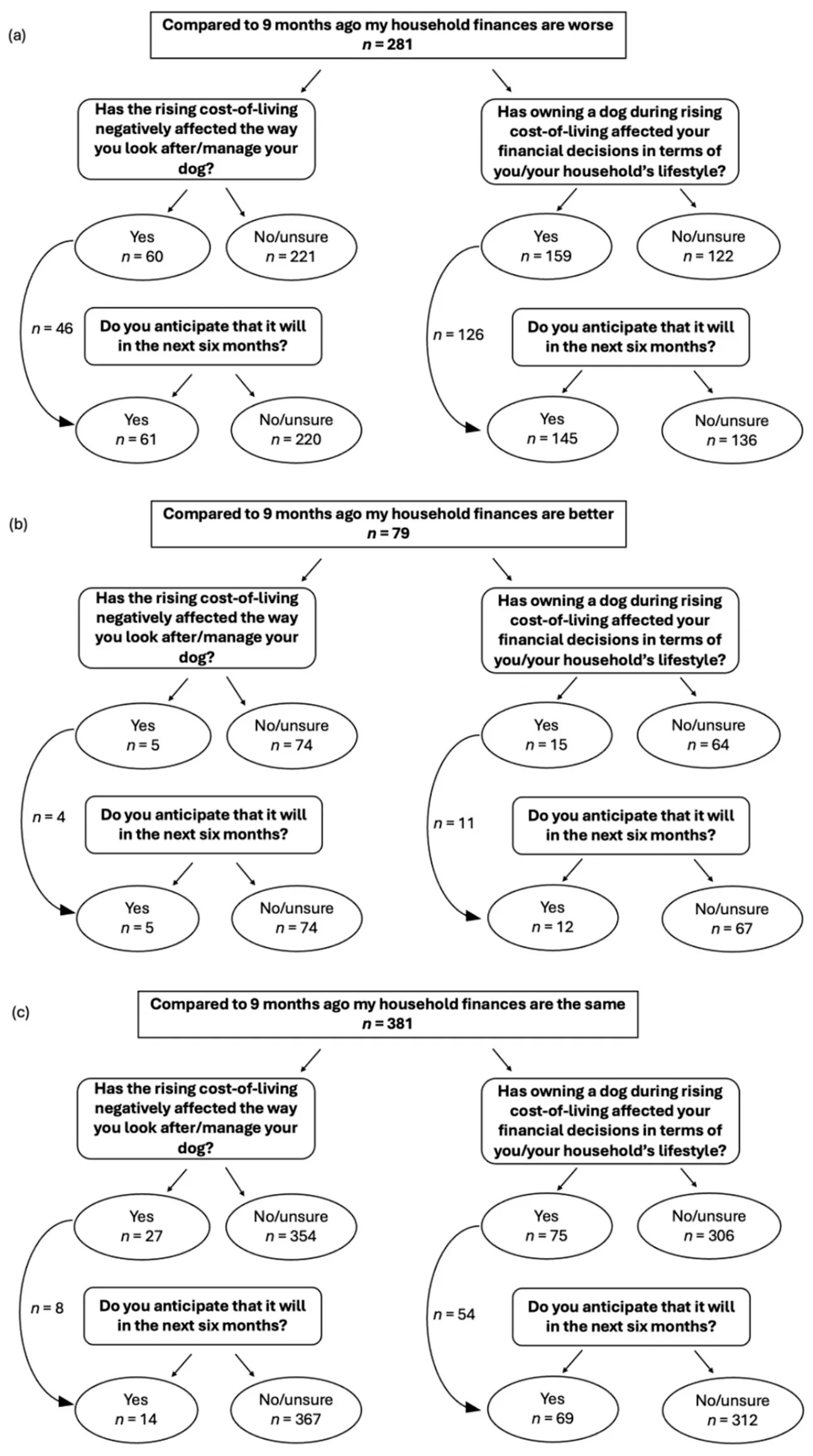
Diagrams to illustrate descriptive responses to questions about dog management and financial decisions of the household by three groups created based on their response to a question about whether their household finances had changed compared to nine months previously. (**a**) *n* = 281 respondents whose household finances were worse. (**b**) *n* = 79 respondents whose household finances were better. (**c**) *n* = 381 respondents whose household finances were unchanged. Total valid responses to the survey, *n* = 770.

Independent of household finances, more respondents reported an impact on their household’s lifestyle (*n* = 249/741; 33.60%) than on their dogs’ management (*n* = 92/741; 12.40%), suggesting that dog management expenditure may be protected relative to household lifestyle expenditure (Figure 3).

Respondents whose household finances had been negatively impacted in the preceding nine months (before survey completion between 23 November 2022 and 4 January 2024) were the most likely to anticipate that their dogs’ management (*n* = 61/261, 23.37%, uOR 6.598, CI 3.84–11.34, *p* < 0.001) and their own household lifestyle (*n* = 81/145, 55.86%, uOR 5.674, 95 CI 4.02–8.01, *p* < 0.001) would be further impacted in the next six months.

Respondents with annual household income ≤ £90,000 were significantly associated with increased odds of reporting that the COLC had had a negative impact upon how they had managed/looked after their dog compared to respondents with an annual household income ≥ £90,001 (<£30,000: uOR 4.056, CI 1.95–8.44, *p* < 0.001; £30,001–£60,000: uOR 2.433, CI 1.22–4.87, *p* = 0.012; £60,001–£90,000: uOR 2.618, CI 1.26–5.44, *p* = 0.010) (Table 1). In contrast, current annual household income was not significantly associated with respondents anticipating that the COLC would have a negative impact upon managing/looking after their dog in the following six months (Table 1).

### 3.5. Impact of COLC on Specific Aspects of Dog Management

Respondents were asked about whether COLC had, or might in the future, impact 14 specific aspects of their dogs’ management. Their responses and the valence of the potential impacts of those changes on individual dog welfare are tabulated below (Table 2).

The majority response to each of the 14 questions was that the COLC had not impacted that aspect of the dog’s management. The aspects of the dogs’ care in which the greatest proportion of respondents reported no COLC-related change were: vaccination (*n* = 738/767; 96.22%); how often and/or for how long the dog would be left at home alone (*n* = 722/757; 95.46%); and decisions related to breeding from an un-neutered dog (*n* = 248/264; 93.94%).

However, in response to every question, some respondents (range: *n* = 29/767; 3.78% (for vaccination)–*n* = 152/766; 19.84% (for insurance)) did report COLC-driven changes. Vaccination was the only aspect of dog management assessed where more respondents reported having made changes which were anticipated to be potentially positive for the dog’s welfare (*n* = 16/767; 2.09%) than negative (*n* = 9/767; 1.17%) or ambiguous (*n* = 4/767; 0.52%).

The aspects of the dogs’ management in which the greatest proportion of respondents reported making or considering making a potentially negative change were decisions related to: attending training classes (*n* = 70/329; 21.23%); insuring their dog (*n* = 127/766; 16.58%); and using day-to-day paid dog care services (*n* = 118/750; 15.73%). Across all aspects of management, the most common potentially negative changes were: being less likely to use a paid dog daycare service (*n* = 113/750; 15.07%); switching to a lower cover/higher excess on their insurance policy (*n* = 68/766; 8.88%); and using a professional groomer less often (*n* = 67/431; 15.55%). Veterinary care-seeking was the aspect of care where the most respondents had made more than one COLC-related potentially negative change (118 changes made by 69 individuals; Table 2).

The aspects of the dogs’ care in which the greatest proportion of responses reported making or considering making a potentially positive change were decisions related to: providing worming treatment (*n* = 35/770; 4.55%); providing flea treatment (*n* = 28/767; 3.65%); and insuring their dog (*n* = 25/776; 3.26%). Within individual response options, the greatest number of individuals making potentially positive changes were: joining a pet health club for worm treatment (*n* = 29/770; 3.77%) or flea treatment (*n* = 26/767; 3.39%) and being more likely to use a paid dog daycare service (*n* = 21/750; 2.80%).

## 4. Discussion

Although existing evidence suggests that financial constraints limit dog owners’ access to care and influence owner decisions [34,40,41,42,43], this is the first quantitative study to investigate a range of specific potential impacts of the UK’s COLC on dog owners’ finances and their ability to afford the cost of care of their dogs between November 2022 and January 2024. Our study population comprised predominantly middle-aged, affluent owners of young “Pandemic Puppy” dogs of innately healthy breeds [45]. Unsurprisingly given these owner demographics, over 60% of respondents reported having been financially unaffected by the COLC. However, one third of respondents (36.26%) indicated that their household incomes were worse in the nine months before the survey was conducted (between 2022 and 2024). This finding suggests that the effects of the COLC may extend beyond traditionally vulnerable populations and may affect dog ownership decisions across a broader socioeconomic spectrum than is often assumed.

Almost three times more respondents reported that COLC had negatively impacted their own household lifestyle compared to their dog’s management. This suggests that many respondents attempted to preserve spending on their dogs’ care over their own lifestyle expenditure, echoing previous findings about the importance of protecting dogs as family members [4,35,36,37]. Despite this, 12.40% of respondents reported that the COLC had negatively impacted their dogs’ care, and 10.80% anticipated that it would in the six months following survey completion.

To facilitate interpretation of the welfare impacts of COLC on dog management, we elected to group our response options to each of the 14 dog management questions into four potential welfare impact subsets: none, potentially positive, potentially negative and ambiguous. This was performed based on the expertise of the authors as veterinary surgeons and animal welfare scientists and on the recent literature. It is acknowledged that different experts may have classified these differently. Whilst most questions incorporated all four response groups, all response options related to neutering and breeding were classed by this research team as ambiguous. This reflects the recent shift to considering the risks versus benefits of neutering for specific individuals of different breeds and genders rather than at a population level [46,47].

Across each of 14 specific facets of dog management assessed, at least 80% of respondents reported no COLC-driven change. However, changes considered likely by these authors to be potentially negative for the dogs’ welfare were most frequently reported in relation to insurance (*n* = 127/766 respondents), use of daytime dog care services (*n* = 118/750), training (*n* = 70/329), prompt access to veterinary advice (*n* = 69/465) and use of paid groomers (*n* = 67/431). Whilst owners may have compensated for these changes by, for example, increasing home grooming or taking on training themselves, these are potentially concerning findings amongst owners of young dogs, and reflect data collected during the same time interval by, and from users of, animal welfare charities (e.g., [4,24]). Whilst opting to reduce the level of insurance cover might feel like a relatively low risk decision in the face of financial pressures with a young and healthy dog, the costs of veterinary care in the UK are widely recognised to be rising [4,15], and the risk of many health disorders increases across the lifespan. Reduced insurance cover may therefore potentially leave owners limited in what veterinary investigations and treatments they can afford for their dog when health problems or injuries do occur, as evidenced by Muldoon and Williams in interviews of owners reflecting on the high costs of veterinary care [4]. Similarly delaying veterinary attention or seeking alternative sources of advice may ultimately lead to higher costs if a problem does not resolve. Owners opting to cancel dog training or seek advice other than from a professional may be risking their dog developing, continuing to have or exacerbating existing behavioural problems which may be challenging to improve [48] and for owners to live with in the longer term [49,50]. This is particularly worrying given the documented high prevalence of owner-reported problem behaviours amongst this cohort of pandemic-acquired dogs [18].

The number of potentially positive changes influenced by the COLC were relatively low, but it was notable that the most common were in relation to increased uptake of vaccination, flea and worming treatment. This suggests that whilst some owners recognised that prevention was likely to be cheaper than treatment if their dog were to be infected or infested, more work still needs to be done to educate dog owners on the benefits of preventive healthcare [29,51,52].

This study has several limitations, predominantly that our respondents were typically more affluent than the average UK resident, which our data suggests may have a protective effect from dog-related impacts of COLC. Consequently, the prevalence of COLC-related changes reported here may underestimate impacts within less affluent sections of the UK dog-owning population. This led to relatively small effect sizes and therefore limited the conclusions we could draw. Additionally, relative household income may not be an accurate reflection of disposable income as the level of essential spending may vary widely between households. In future research, adoption of the recently developed demographic questions for collecting equity-relevant data [53] may be preferable, specifically asking individuals how easily they can afford their basic living expenses rather than focusing on income. As this study had no prior hypothesis and was exploratory in nature, no multivariable modelling was performed to account for confounding, and the results should be interpreted with this in mind.

All responses were reliant on self-report and as such are likely to have included an unmeasurable degree of recall bias. This survey was part of a longitudinal cohort study, so all respondents had previously completed surveys about this dog and their relationship with them; for this individual survey the response rate was the lowest of the series at less than a third. These participants may therefore have been particularly bonded to their dogs or committed to their care, which may have impacted the decisions they made in relation to the COLC differently to the wider UK population. By using this cohort, selection and survivorship biases will have been introduced, favouring those owners able and motivated to voluntarily participate in our ongoing research. Furthermore, this “Pandemic Puppies” cohort is known to differ from other UK dogs in relation to their ownership, methods of acquisition and behaviours [7,8,18,54]. This potentially limits generalisability of these results to the wider UK dog and owner populations. Animal welfare was not assessed directly, and inferences are based only on owner-reported management decisions. Our classification of impacts into positive, negative and ambiguous, whilst created by consensus, is subjective, and others may have classified the same responses in a different way. The literature on which these classifications were based was reviewed narratively rather than systematically. Whilst we asked participants whether any changes they made were specifically influenced by the COLC, it must be acknowledged that some of the changes made may have happened anyway, reflecting an unmeasured baseline of change within dog management decision-making over time.

## 5. Conclusions

Our findings suggest that owners of at least 10% of this cohort of UK dogs purchased during the COVID-19 pandemic reported management changes perceived by these authors as potentially detrimental to their dogs’ welfare, with no owner income or age bracket completely protected. As a result of financial pressures, owners report reducing their dogs’ insurance cover and cancelling or deferring visits to professional dog trainers, groomers, day care providers and veterinary practices. Since the COLC has continued into 2026 and given the relative affluence of our cohort, it is likely that our report represents an underestimate of the negative impacts of the COLC on canine welfare.

## Figures and Tables

**Table 1:** Descriptive and univariable binary logistic regression analysis of impacts of the ‘Cost-of-Living Crisis’ (COLC) amongst UK-based owners of 36-month-old dogs purchased under 16 weeks of age between 23 March and 31 December 2020 in terms of annual household income. The Likert options of “Very much”, “Quite a lot” and “A little bit” were combined to give the outcome of ‘Yes’. The response options “I’m not sure” and “I’d rather not say” are not included in the analysis. * 95% confidence interval. uOR = unadjusted odds ratio. Significant results in the table are formatted in bold font. Total valid responses to the survey, *n* = 770.

Variable	Income	*n* Not at All; Yes	% Yes	uOR	95. % CI *	*p*-Value
COLC has negatively affected looking after/managing their dog (*n* = 647)	**≤£30,000**	**86; 26**	**23.2**	**4.056**	**1.950–8.437**	**<0.001**
	**£30,001–£60,000**	**182; 33**	**15.3**	**2.433**	**1.215–4.869**	**0.012**
	**£60,001–£90,000**	**123; 24**	**16.3**	**2.618**	**1.260–5.441**	**0.010**
	≥£90,001	161; 12	6.9	Reference category		
COLC is anticipated to_negatively affect looking after/managing their dog (*n* = 606)	≤£30,000	85; 15	15.0	1.916	0.883–4.160	0.100
	£30,001–£60,000	181; 25	12.1	1.500	0.753–2.986	0.249
	£60,001–£90,000	114; 20	14.9	1.905	0.923–3.932	0.081
	≥£90,001	152; 14	8.4	Reference category		

**Table 2:** Descriptive analysis of impacts of the ‘Cost-of-Living Crisis’ (COLC) amongst UK-based owners of 36-month-old dogs purchased under 16 weeks of age between 23 March and 31 December 2020 on specific aspects of dog maintenance. * Respondents could choose > 1 answer to this question. Total valid responses to the survey, *n* = 770.

Health/Lifestyle Question	Response Option	Total Responses (% per Respondent)	Potential Impact on Animal Welfare	Respondents per Welfare Impact Category (% per Respondent)
Health: Has/will the rising cost-of-living affect decisions regarding insuring your dog? (*n* = 766)	No change	614 (80.16)	None	614 (80.16)
	Increased level of insurance cover	12 (1.56)	Potentially positive	25 (3.26)
	Will now insure having not insured previously	13 (1.70)		
	Cancelled/cancelling policy	20 (2.61)	Potentially negative	127 (16.58)
	Will not renew	10 (1.30)		
	Reduced level of cover	29 (3.79)		
	Switched to lower cover/higher excess	68 (8.88)		
Health: Has/will the rising cost-of-living affect decisions regarding vaccinating your dog? (*n* = 767)	No change	738 (96.22)	None	738 (96.22)
	Will now vaccinate having not done so recently	3 (0.39)	Potentially positive	16 (2.09)
	Will ensure my dog’s vaccines are up to date	13 (1.69)		
	Will no longer vaccinate	9 (1.17)	Potentially negative	9 (1.17)
	Used to titre test, will now just vaccinate	4 (0.52)	Ambiguous	4 (0.52)
Health: Has/will the rising cost-of-living affect your decisions regarding the brand, source or use of regular worm treatments for your dog? (*n* = 770)	No change	632 (82.07)	None	632 (82.07)
	Will now join a veterinary pet health club	29 (3.77)	Potentially positive	35 (4.55)
	Will now re-start worming	6 (0.78)		
	Will no longer worm	6 (0.78)	Potentially negative	38 (3.64)
	Will now worm less often	22 (2.86)		
	Will no longer buy via a veterinary pet health plan	10 (1.30)		
	Will now buy from somewhere other than the vets	65 (8.44)	Ambiguous	65 (8.44)
Health: Has/will the rising cost-of-living affect your decisions regarding the brand, source or use of regular flea treatments for your dog? (*n* = 767)	No change	634 (82.66)	None	634 (82.66)
	Will now join a veterinary pet health club	26 (3.39)	Potentially positive	28 (3.65)
	Will now start treating having not done so recently	2 (0.26)		
	Will no longer treat	7 (0.91)	Potentially negative	43 (5.60)
	Will now use flea products less often	24 (3.13)		
	Will no longer buy via a veterinary pet health plan	12 (1.56)		
	Will now buy from somewhere other than the vets	62 (8.08)	Ambiguous	62 (8.08)
* Health: In the last 9 months, has there been a change in the diet you typically feed your dog, including the type, source or brand, that is due in part or entirely to the rising cost-of-living? (respondent *n* = 763; response *n* = 778)	No change	548 (71.82)	None	656 (85.98)
	Change but not related to the cost-of-living rising	108 (13.79)		
	Changed to a cheaper brand	60 (7.86)	Potentially negative	71 (9.31)
	Changed to a cheaper type of diet	14 (1.83)		
	Changed to a cheaper source of the same diet	53 (6.9)	Ambiguous	53 (6.95)
Health: Given that your dog is not neutered, has/will the rising cost-of-living affect your plans regarding neutering? (*n* = 265)	No change	237 (89.43)	None	237 (89.43)
	Was planning to neuter but will now breed	0 (0.00)	Ambiguous	28 (10.57)
	Have cancelled plans to neuter but no plan to breed	8 (3.02)		
	Now plan to neuter	5 (1.89)		
	Have now neutered my dog	6 (2.26)		
	Not sure	9 (3.40)		
Health: Given that your dog is not neutered, has/will the rising cost-of-living affect your decisions regarding breeding your dog? (*n* = 264)	No change	248 (93.94)	None	248 (93.94)
	Now planning to breed where not planning before	1 (0.38)	Ambiguous	16 (6.06)
	Was planning to breed but now plan not to	15 (5.68)		
* Health: Given that your dog had a health problem in the previous 9 months, has the rising cost-of-living affect your plans regarding veterinary seeking decisions and actions in the last 9 months? (respondent *n* = 465; response *n* = 559)	No change	396 (85.16)	None	419 (90.11)
	No but may change in the future	23 (4.95)		
	Now qualify for charity veterinary care	1 (0.21)	Potentially positive	1 (0.21)
	Delayed seeing a vet to see if a problem improves by itself	44 (9.46)	Potentially negative	69 (14.84)
	Spending more time looking online before seeking veterinary advice	36 (7.74)		
	Using more home remedies	22 (4.73)		
	Have not taken the dog to the vets for a minor problem	8 (1.72)		
	Have cancelled or delayed a planned visit	8 (1.72)		
	Using online consultations included with pet insurance as well as/instead of own vet	14 (3.01)	Ambiguous	21 (4.52)
	Using cheaper vet practice/shopping around on price	5 (1.08)		
	Not sure	2 (0.43)		
* Lifestyle: Given that you had considered attending training with your dog, has/will the rising cost-of-living affect any aspect of your dog’s training, for example, attending training classes? (respondent *n* = 329; response *n* = 375)	No change	187 (56.84)	None	272 (82.67)
	No, still prefer training advice from a professional	117 (35.56)		
	Won’t buy or have cancelled planned training	27 (8.20)	Potentially negative	70 (21.23)
	Less likely now/in the past to seek professional training advice	44 (13.37)		
* Lifestyle: Has the rising cost-of-living affected your plans regarding behavioural advice seeking decisions and actions in the last 9 months? (respondent *n* = 762; response *n* = 820)	My dog has not had a behavioural problem	509 (66.80)	None	693 (90.95)
	No change	176 (23.10)		
	No change yet but may in the future	23 (3.01)		
	Used a charity behaviour helpline	5 (0.66)	Potentially positive	5 (0.66)
	Delayed seeking advice to see if it improved by itself	16 (2.10)	Potentially negative	67 (8.79)
	Did not seek advice where would have done so previously	10 (1.31)		
	Used free online advice rather than seeing a behaviourist	4 (0.52)		
	Already cancelled a planned visit to a behaviourist	0 (0.00)		
	Asked friends and family first	12 (1.57)		
	Asked a dog trainer first	10 (1.31)		
	Shopped around on price	2 (0.26)		
	Spent more time looking online first	43 (5.64)		
	Not sure	10 (1.31)	Ambiguous	10 (1.31)
Lifestyle: Given that you have used a dog groomer in the past, has/will the rising cost-of-living affect your plans regarding your dog’s professional grooming? (*n* = 431)	No change	363 (84.22)	None	363 (84.22)
	Have/will use a professional groomer more often	1 (0.23)	Potentially positive	1 (0.23)
	Have/will use a professional groomer less often	67 (15.55)	Potentially negative	67 (15.55)
Lifestyle: Has/will the rising cost-of-living affect your plans regarding your use of paid services to help with your dog’s day-to-day care (e.g., dog walkers, doggy daycare)? (*n* = 750)	Have never used paid daycare	462 (61.60)	None	611 (81.47)
	No change	149 (19.87)		
	More likely to use a paid service	21 (2.80)	Potentially positive	21 (2.80)
	Less likely to use a paid service	113 (15.07)	Potentially negative	118 (15.73)
	Use cheaper paid service	5 (0.67)		
Lifestyle: Given that you have used others to look after your dog when on holiday before, how has/will the rising cost-of-living affect your decision to leave your dog and/or go away on holiday? (*n* = 648)	No change	545 (84.10)	None	545 (84.10)
	Switch to cheaper paid service	5 (0.77)	Potentially negative	26 (4.01)
	Switch to family or friends	21 (3.24)		
	Can’t afford a holiday	6 (0.93)	Ambiguous	77 (11.88)
	Going on a cheaper holiday to cover dog’s care	6 (0.93)		
	Dog will now holiday with me	65 (10.03)		
Lifestyle: Has/will the rising cost-of-living affect the amount of time/how often your dog is left at home alone? (*n* = 757)	No change	722 (95.46)	None	722 (95.46)
	Dog will be left alone for less time/less often	9 (1.18)	Potentially positive	9 (1.18)
	Dog will be left alone for more time/more often	26 (3.43)	Potentially negative	26 (3.43)

## Data Availability

The data presented in this study are available on request from the corresponding author.

## Supplementary Materials

The following supporting information can be downloaded at: https://www.mdpi.com/article/10.3390/ani16172656/s1. File S1: Survey; File S2: Supplementary Tables. References [4,55,56,57,58,59,60,61,62,63,64,65,66,67,68,69,70,71,72,73,74,75,76,77,78] are included in the Supplementary Materials.
