# Supplementary material for: Impacts of the ‘Cost-of-Living Crisis’ on UK-Based Owners of Pandemic Puppies
Source: Animals (Basel). 2026 Aug 24;16(17):2656. doi: 10.3390/ani16172656 (PMC13564328; doi:10.3390/ani16172656)
Supplement: Supplementary file 1 [file animals-16-02656-s001.zip › File S1.pdf]

Supplementary File: Full Questionnaire Text

CONSENT QUESTIONS AND INCLUSION CRITERIA:

Pandemic Puppies Follow-Up Survey, 36 Months – Exploring the "Cost-of-Living Crisis"

In November-December 2020, you were one of 7545 caring owners who kindly responded to a survey from our Pandemic Puppies team at the Royal Veterinary College (RVC). This research has already led to two peer-reviewed papers (paper 1, paper 2) and is majorly changing how we all understand the serious impact on dog welfare that resulted from the COVID-19 pandemic – thank you!

At that time, you generously indicated your willingness for you and your dog to be involved in further research aiming to improve dog welfare. As a result, you have been specifically chosen to take part in our next exciting research project that will follow the lives of puppies bought during the 2020 phase of the COVID-19 pandemic even further into the future. We would be really grateful if you would complete a further survey now that your dog is around 36 months of age.

If you no longer have the dog about which you completed the previous survey(s), we would still like to hear from you; learning more about the reasons for this change will be really helpful to our research team and will help other dogs and owners in the future. You will be able to indicate that you no longer have your dog at the very start of the survey which will then take no more than 5 minutes to complete.

If you still own your dog but you do not wish to hear from us anymore, please fill in the very start of the survey to indicate this; following this you will not receive any further contact from the Pandemic Puppies research team here at the RVC.

What is this further research focused upon?

This new research, generously funded by Battersea Dogs and Cats Home, aims to better understand and support the needs of owners who, like you, who bought a puppy during the COVID-19 pandemic, and are now raising them during the so-called "cost-of-living crisis". By taking part in this survey, animal welfare organisations and charities will have a better understanding of your unique experiences and challenges, enabling them to provide better support for other owners and dogs right across the UK.

What does the study involve?

We are inviting you to take part in this further survey now that your dog has reached 36 months of age. This survey will explore some longer-term impacts following buying a puppy during the COVID-19 pandemic. The survey will specifically focus upon dog health, behaviour and dog-owner relationships, as well as exploring any potential impacts upon your dog, and yourself as their owner,

of the currently increasing cost-of-living in the UK. If you have completed any of our previous surveys as your dog reached 21, 24 and/or 27 months of age, some of this content will be familiar to you, and we will use your new responses to this survey to track changes over time for owners and dogs. The section on the rising cost-of-living is new, and we would appreciate responses regardless of the level of impact that you feel these price changes have had upon your household.

To take part, you simply answer a series of questions online. The survey will take no longer than 30-40 minutes (or under 5 minutes if you are opting out/no longer have the dog you previously completed a survey(s) for) and you are free to answer the questions across more than one session. The link you have been provided with in the accompanying email is unique to you and your dog and should not be shared for use by others. You will have 28 days from receiving the first email to complete the survey, after which time your unique link will expire.
We ask once again that you answer the survey for the same dog that you answered the initial survey for in November-December 2020. As a reminder, if you bought more than one puppy between 23 March 2020 and 31 December 2020, you were asked to answer for the youngest puppy. If two puppies of the same age were purchased (e.g., littermates), you were asked to answer for the puppy whose name came first alphabetically.

The survey is ideally to be completed by the person who was/is most involved in the care of this dog. If possible, this would be the same person who completed the initial survey in autumn/winter 2020 and provided us with this email address. If that is not possible, we are happy for the survey to be completed by another person involved in the daily care of this dog.

How do I complete the survey?

Once you have started the survey, you are able to save your responses so that you can answer the questions across more than one session using your device of choice (e.g., smartphone, PC) until you submit your final survey answers to us. After submission, you will no longer be able to access your responses. To pause the survey and return later, simply press the 'Save and Return Later' button and you will be provided with an auto-generated return code, which you will be required to enter upon returning to the survey in order to continue. **If you accidently press the 'back' button on your browser whilst completing the questions and lose the survey, it can be reloaded by refreshing the page or pressing F5.**

What will happen to my information?

Your information will be kept securely and confidentially by the RVC in accordance with GDPR. Only the scientists undertaking this study (Dr. Rowena Packer, Dr. Dan O'Neill, Dr. Zoe Belshaw, Dr. Claire Brand, Dr. Camilla Pegram, Dr. Fiona Dale, Dr. Kim Stevens and student members of their team) will have access to your answer data. This data will be anonymised and used only for research. Only anonymised results and data will be published. Publications may include quotes from your written responses, but these will be anonymised, and you will not be identifiable from any quotes.

This study has received ethical approval from the RVC Social Science Research Ethical Review Board (SR2020-0259). If you have any further questions about the study, please contact Dr. Rowena Packer and Dr. Claire Brand (pandemicpuppies@rvc.ac.uk).

Opting out

You can opt out of this survey at any time. Data you have already submitted to us remains with us, but we do not contact you regarding further involvement in the study, and do not send you any further reminders for this survey. There is an option to do this at the beginning of this survey, in addition to the option to be removed from our mailing list which we use to send Newsletters to update participants about project news and publications.

Can I change my mind and withdraw my data?

Should you wish to withdraw your data from the study at any point, please contact Dr. Rowena Packer and Dr. Claire Brand (pandemicpuppies@rvc.ac.uk). Any data that you request to be withdrawn will be permanently deleted.

What if taking part raises concerns?

If this study raises any concerns about your dog's welfare, then please contact a veterinary surgeon to discuss. Links on how to find a veterinary surgeon in your area are included at the end of the survey, in addition to links for free/subsidised veterinary care. Resources on specific canine welfare issues raised are also be included at the end of the survey.

If this study raises any concerns about your own mental health or well-being, then please access these NHS resources:
www.nhs.uk/conditions/stress-anxiety-depression/mental-health-helplines/

Q1
I confirm that:

1. I am over 18 years of age.
2. I am a resident of the UK.
3. I have read and understood the above information and give consent for my answers to be used for this research study and any resulting publications.

ESSENTIAL QUESTIONS (ALL PARTICIPANTS):

Q2

Do you still own the dog you told us about in the 2020 'Pandemic Puppies' survey (that you purchased between 23rd March-31st December 2020)?

N.B. As a reminder, if you purchased more than one puppy in the time period above, we asked you to answer for the youngest dog, or in the case of littermates, the dog whose name came first alphabetically.

1. Yes – I still have my dog **[SURVEY LOGIC – skip questions 3-15]**
2. No – I have rehomed/sold/given away this dog [**SURVEY LOGIC – skip questions 8-14]**
3. No – my dog has died/been put to sleep [**SURVEY LOGIC – skip questions 3-7 and 13-14]**
4. No – my dog was stolen/lost and is still missing **[SURVEY LOGIC – skip questions 3-12]**

Q3

If you are comfortable doing so, please describe in your own words why you decided to rehome, sell, give away or otherwise stop owning this dog. **[free text]**

Q4

Which month/year did you rehome, sell or give away this dog?

Drop down month list

Drop down year list (2020-2023)

Q5

If you are happy to share with us, please specify how you rehomed, sold or gave away this dog?

1. Rehomed via a multi-breed, multi-location rescue charity (e.g., Dogs Trust, RSPCA, Battersea, Blue Cross, Wood Green, Jerry Green)
2. Rehomed via a multi-breed rescue organisation not listed above (e.g., local, independent, and/or single-site rescue centres)
3. Rehomed via a breed specific rescue (e.g., English Springer Spaniel Welfare, Border Collie Trust GB, Doodle Trust)
4. Rehomed/given to a family member free of charge
5. Rehomed/given to a friend free of charge
6. Rehomed/given to someone I did not know previously free of charge
7. Privately sold to a new owner via an internet selling site (e.g., Pets4Homes)
8. Privately sold to a new owner I already knew (e.g., friend, family, acquaintance)
9. Other (please specify below) **[free text]**

Q6

Was the rising cost-of-living a factor in this decision?

N.B. If you would like to explain your answer further in your own words, please feel free to do so below. **[free text]**

1. Yes, entirely
2. Yes, partly

3. No, not at all
4. I’m not sure

Q7

Please share any other thoughts or feelings about your experiences of buying a dog during the pandemic and subsequently rehoming, giving away or selling them, including the reasons why, if you feel comfortable to do so. **[free text]**

Q8

We’re sorry to hear that your dog is no longer alive. If you feel comfortable doing so, we’d like you to share a bit more about what happened to enable us to help other owners and their dogs. Firstly, how did your dog die?

1. Put to sleep (euthanised) **[SURVEY LOGIC – skip question 10]**
2. Natural or unassisted death (not euthanised/put to sleep) **[SURVEY LOGIC – skip question 9]**
3. Accidental death (e.g., run over) **[SURVEY LOGIC – skip question 9]**

Q9

If you are comfortable doing so, please describe in your own words what led to the decision for your dog to be put to sleep, including whether the rising cost-of-living was a factor. **[free text]**

Q10

If you are comfortable doing so, please describe in your own words what circumstances led to your dog's death. **[free text]**

Q11

Please indicate which month/year your dog was put to sleep or passed away.
Drop down month list
Drop down year list (2020-2023)

Q12

Would you like to share any other thoughts or feelings about your experiences of buying a dog during the pandemic and subsequently losing them? **[free text]**

Q13

We’re sorry to hear that your dog has been stolen/gone missing. If you feel comfortable doing so, we’d like to ask you to answer a few questions about what happened. Firstly, please indicate which month/year your dog was stolen/went missing.
Drop down month list
Drop down year list (2020-2023)

Q14

Would you like to share any thoughts or feelings about your experiences of buying a dog during the pandemic and them subsequently being stolen/going missing? **[free text]**

Thank you for giving your time to explain why you no longer have your dog.
Please indicate below whether you would still be interested in receiving project updates.

Q15

Would you like to receive project updates from the RVC about the Pandemic Puppies study?

1. Yes, I would like to receive updates about the research you are carrying out (please do not delete my contact details) **[SURVEY LOGIC – send to the end of the survey, debrief section 2]**
2. No, I would rather not hear from you again in the future (please delete my contact details) **[SURVEY LOGIC – send to the end of the survey, debrief section 2]**

Q16

Are you willing to take part in this survey about your experiences with your dog?
This would greatly help our overall understanding of dog welfare and enable us to help improve the lives of dogs in the future.
N.B. This survey will take ~30-40 minutes and is to be completed within 28 days of the initial link being sent via email. You can answer the questions in more than one session.

1. Yes, I would like to take part in the current research about my dog
2. No, but I would like to receive updates and possibly take part in future research you are carrying out (please do not delete my contact details) **[SURVEY LOGIC – send to the end of the survey, debrief section 1]**
3. No, I would rather not take part in further research about my dog and would prefer not to hear from you again in the future (please delete my contact details) **[SURVEY LOGIC – send to the end of the survey, debrief section 1]**

Q17

If you are happy to do so, please tell us your dog's name. **[free text]**

THE COST-OF-LIVING CRISIS QUESTIONS (PARTICIPANTS WHO STILL OWN THEIR DOG):

In this section we would like to understand whether and how the rising cost-of-living is impacting or may impact in the future upon you/your household and your dog. We would appreciate your responses to these questions regardless of the level of impact you feel changes to cost-of-living in the UK have had or will have upon you/your household.

Q18

Has the rising cost-of-living affected your decisions and actions regarding seeking advice for your dog's health from a veterinary surgeon in the last 9 months?

Please select **all options** that apply.

1. N/A – my dog has not had any health issues in this time period
2. No
3. No, not yet but I think it will in the future
4. I'm not sure
5. Yes, I've delayed seeking advice to see if a health issue got better by itself
6. Yes, I've spent more time finding information about my dog's health issues online than I would otherwise have done so
7. Yes, I've tried home remedies/suggestions from non-vets first before going to the vets
8. Yes, I've not yet taken my dog for a minor problem (e.g., a new lump), whereas in the past I would have already done so
9. Yes, I've cancelled/delayed a planned visit, investigation, operation or dental
10. Yes, I've started using free online consultations linked to my pet insurance instead of/as well as going to the vets
11. Yes, I've changed to a cheaper veterinary practice/shopped around on price
12. Yes, I now qualify for charity veterinary care (e.g., PDSA, Blue Cross, etc.)
13. Other (please specify below) **[free text]**

Q19

Has/will the rising cost-of-living affect decisions regarding insuring your dog?

1. Yes, I have reduced/will reduce my level of cover to make it cheaper
2. Yes, I have switched/will switch companies to a lower premium/higher excess
3. Yes, I have cancelled/will cancel their insurance
4. Yes, I have not/plan not to renew their insurance
5. Yes, I have increased/will increase my insurance level of cover
6. Yes, I never insured my dog in the past, but have/will do so now
7. No

Q20

Has/will the rising cost-of-living affect decisions regarding vaccinating your dog?

1. Yes, I never vaccinated my dog in the past but do/will do so now

2. Yes, I vaccinated my dog in the past, but will no longer do so
3. Yes, my dog's vaccines are not up to date, but I will now ensure they are
4. Yes, I usually titre test but will now just vaccinate
5. No
6. Other (please specify below) **[free text]**

Q21

Has/will the rising cost-of-living affect your decisions regarding the brand, source or use of regular worm treatments for your dog?

1. Yes, I have started/will now start worming my dog
2. Yes, I have not wormed/will no longer worm my dog
3. Yes, I have bought/will now buy wormer from somewhere other than the vets (e.g., to a cheaper source)
4. Yes, I used to subscribe to a pet health club to pay for worm treatments via my vets but have stopped/plan to stop this
5. Yes, I have started to/plan to subscribe to a pet health club to pay for worm treatments via my vets
6. Yes, I do/will worm my dog less often
7. No
8. Other (please specify below) **[free text]**

Q22

Has/will the rising cost-of-living affect your decisions regarding the brand, source or use of regular flea treatments for your dog?

1. Yes, I have started/will now start flea treating my dog
2. Yes, I have not/will no longer flea treat my dog
3. Yes, I have bought/will now buy flea treatments from somewhere other than the vets (e.g., to a cheaper source)
4. Yes, I used to subscribe to a pet health club to pay for flea treatments via my vets but have stopped/plan to stop this
5. Yes, I have started to/plan to subscribe to a pet health club to pay for flea treatments via my vets
6. Yes, I do/plan to flea treat my dog less often
7. No
8. Other (please specify below) **[free text]**

Q23

Has/will the rising cost-of-living affect your decisions regarding neutering your dog?

1. N/A – my dog is already neutered **[SURVEY LOGIC – skip question 24]**
2. Yes, I have now had my dog neutered
3. Yes, I now plan to neuter my dog
4. Yes, I have cancelled plans to neuter my dog, but don't plan to breed from them

5. Yes, I have cancelled plans to neuter my dog, and now plan to breed from them
6. I'm not sure
7. No, it has not affected my decision

Q24

Has/will the rising cost-of-living affect your decisions regarding breeding your dog?

1. N/A – my dog was already neutered prior to the rising cost-of-living
2. Yes, I wasn't planning to breed from my dog, but now plan to breed from them
3. Yes, I was planning to breed from my dog, but now plan not to breed from them
4. No, it has not affected my decision

Q25

Has/will the rising cost-of-living affect any aspect of your dog's training, for example, attending training classes?

N.B. Professional advice refers advice provided by a dog trainer, dog behaviourist or veterinary nurse/veterinary surgeon.

Please select **all options** that apply.

1. N/A – I don't attend any training classes with my dog
2. No, it has not affected any training class attendance plans
3. No, I still prefer to seek professional advice when it comes to any training issues
4. Yes, I have not/will not purchase or have cancelled/plan to cancel training classes (in person/online) that I had previously wanted to attend
5. Yes, I have been/will be less likely seek professional advice for training issues

Q26

Has the rising cost-of-living affected whether or how you access advice regarding any specific behaviour problems you have experienced with your dog in the last 9 months?

N.B. Behaviour professional refers to a qualified dog behaviourist or veterinary nurse/veterinary surgeon.

Please select **all options** that apply.

1. N/A – my dog has not had any behavioural problems in this time period
2. No
3. No, not yet but I think it will in the future
4. I'm not sure
5. Yes, I've delayed seeking advice to see if a behaviour problem got better by itself
6. Yes, I've not sought advice from a behaviour professional, whereas in the past I would have done so
7. Yes, I've started using free online consultations linked to my pet insurance instead of/before seeking referral to a behaviour professional in person
8. Yes, I've cancelled a planned visit to see a behaviour professional regarding my dog's behaviour problem(s)
9. Yes, I've tried suggestions from non-professionals (e.g., friends/family, etc.) instead of/before seeking referral to a behaviour professional

10. Yes, I’ve tried suggestions from a dog trainer instead of/before seeking referral to a behaviour professional
11. Yes, I’ve changed to a cheaper behaviour professional/shopped around on price
12. Yes, I’ve used charity behaviour helplines (e.g., Dogs Trust)
13. Yes, I've spent more time finding information about my dog's behaviour problems online than I would otherwise have done so

Q27

In the last 9 months, has there been a change in the diet you typically feed your dog, including the type, source or brand, that is due in part or entirely to the rising cost-of-living?
Please select **all options** that apply.

1. Yes, a change to a cheaper brand
2. Yes, a change in where I purchase my dog’s food, to a cheaper source (e.g., from in store to online, from your vets to an online company, or to a different online source)
3. Yes, a change from one general type of diet to another cheaper type of diet (e.g., raw diet to a complete dry diet, a prescription diet to non-prescription diet, etc.)
4. Yes, a change but not related to the rising cost-of-living
5. No change
6. Other (please specify below) **[free text]**

Q28

Has/will the rising cost-of-living affect how often or whether your dog is professionally groomed?

1. N/A – my dog has never been professionally groomed
2. Yes, my dog is being/will be professionally groomed less often
3. Yes, my dog is being/will be professionally groomed more often
4. No change

Q29

Has/will the rising cost-of-living affect your decisions regarding extra help with the day-to-day care of your dog, in a paid capacity (i.e., use of dog walkers, doggy daycare, etc.)?

1. Yes, I am less likely to use/have stopped using paid services
2. Yes, I am more likely to use/have started using paid services
3. N/A – I have never needed to use paid services to help to look after my dog
4. Other (please specify below) **[free text]**

Q30

In the past, have you paid to leave this dog with a professional dog sitter and/or put them in boarding kennels when you went on holiday?

1. Yes
2. No
3. I’m not sure/can’t remember
4. N/A – I have not gone on holiday since owning this dog

Q31

Thinking about potential/booked holidays, and factoring in the cost of boarding kennels, dog sitters, or other options of caring for your dog whilst you are away, has/will the rising cost-of-living affect your decision to leave your dog behind if/when you go away?

1. No **[SURVEY LOGIC – skip question 32]**
2. I'm not sure **[SURVEY LOGIC – skip question 32]**
3. Yes
4. N/A – I have no booked/planned holidays **[SURVEY LOGIC – skip question 32]**

Q32

How has/will the rising cost-of-living affect your decision to leave your dog and/or go away on holiday?

1. I have gone/will now go on holidays where I can take my dog with me
2. I have gone/will go on a cheaper holiday so I can afford the cost of care for my dog
3. I have switched/will switch to using a cheaper paid service to care for my dog whilst I am away
4. I have stopped using/will no longer be able to use paid services at all (e.g., boarding kennels, dog sitters, etc.) and so do/will use an unpaid service from friends/family instead
5. I have been/will now be unable to go on holidays at all because of the cost of care for my dog
6. Other (please specify below) **[free text]**

Q33

Has/will the rising cost-of-living affect the amount of time/how often your dog is left at home alone?

1. Yes, my dog will be left alone longer/more often
2. Yes, my dog will be left alone for less time/less often
3. No – there will be no change in how long/often my dog is left alone as a result of rising cost-of-living
4. Other (please specify below) **[free text]**

Q34

Compared to 9 months ago, have your household finances changed?

1. My household finances are the same
2. My household finances are worse
3. My household finances are better
4. I'd rather not say
5. I'm not sure

Q35

Has the rising cost-of-living negatively affected the way you look after/manage your dog?

N.B. If you would like to explain your answer further in your own words, please feel free to do so below. **[free text]**

1. Very much
2. Quite a lot
3. A little bit
4. Not at all
5. I'm not sure
6. I'd rather not say

Q36

Do you think the rising cost-of-living will negatively affect the way you look after/manage your dog in the next 6 months?

N.B. If you would like to explain your answer further in your own words, please feel free to do so below. **[free text]**

1. Very much
2. Quite a lot
3. A little bit
4. Not at all
5. I'm not sure
6. I'd rather not say

Q37

Has owning a dog during rising cost-of-living affected your financial decisions in terms of you/your household's lifestyle?

N.B. If you would like to explain your answer further in your own words, please feel free to do so below. **[free text]**

1. Very much
2. Quite a lot
3. A little bit
4. Not at all
5. I'm not sure
6. I'd rather not say

Q38

Do you think owning a dog during rising cost-of-living will affect your financial decisions in terms of you/your household's lifestyle in the next 6 months?

N.B. If you would like to explain your answer further in your own words, please feel free to do so below. **[free text]**

1. Very much
2. Quite a lot
3. A little bit
4. Not at all
5. I'm not sure
6. I'd rather not say

Q39

Do you foresee any changes to your/your household's circumstances/finances in the next 6 months that might make it easier or more difficult to continue owning your dog?

1. No **[SURVEY LOGIC – skip question 40]**
2. Yes – changes that might make it easier
3. Yes – changes that might make it more difficult, e.g., cost of living
4. I'm not sure
5. I'd rather not say **[SURVEY LOGIC – skip question 40]**

Q40

Please describe in your own words any anticipated changes to your/your household's circumstances/finances in the next 6 months that might make it easier or more difficult to continue owning your dog. **[free text]**

DEMOGRAPHIC QUESTIONS (PARTICIPANTS WHO STILL OWN THEIR DOG):

In this section we would like to understand how your living circumstances may have changed since you purchased your dog.

We are including some questions about your/your household finances and asking for your postcode (both of which are entirely voluntary).

The reason we are doing this is to assess which dog owners are being most impacted by the rising cost-of-living, and therefore need support, on a regional basis.
Your postcode information will not be analysed at an individual level but across the whole population, you will not therefore be identifiable by giving this information.

Some of these questions are very similar to those asked in previous surveys, but for some owners the answers may have changed.

Taking the time to answer these questions will greatly help our overall understanding of dog and owner welfare in the face of the rising cost-of-living and enable us to help improve the lives of both owners and dogs in the future.

Q41

Have you had any changes to the number of dogs in your household since the first survey (in November/December 2020)?

1. No – no changes **[SURVEY LOGIC – skip question 42]**
2. Yes – I now own fewer dogs
3. Yes – I now own more dogs
4. Yes – I now own the same number of dogs, but there has been a change in which dogs I own

Q42
Why has there been a change to the number of dogs in your household since the first survey (in November/December 2020)?
Please select **all options** that apply.

1. A dog(s) in the household died due to old age
2. A dog(s) in the household died for a reason unrelated to old age
3. I sold a dog(s)
4. I rehomed a dog(s) to another household/rescue organisation
5. A dog(s) was stolen/lost
6. I purchased a dog(s)
7. I adopted/rescued a dog(s) into my household

Q43
Have you considered adding another dog(s) to your household since the first survey (in November/December 2020), whether by purchasing/adopting a puppy or rehoming/rescuing an older dog, but have been put off due to the rising cost-of-living?

1. Yes
2. No

Q44
What best describes your current living situation?
N.B. This question is very similar to that asked in the first survey in November/December 2020, but for some participants their answer may have changed since that time.

1. Live alone **[SURVEY LOGIC – skip question 45]**
2. Live in an adult-only home (over 18 years old) **[SURVEY LOGIC – skip question 45]**
3. Live in a home with adults and children
4. Live in a home with children where I am the only adult
5. Other (please specify) **[free text] [SURVEY LOGIC – skip question 45]**

Q45
How old are the children that your dog currently shares a home with?
N.B. This question is very similar to that asked in the first survey in November/December 2020, but for some participants their answer may have changed since that time.
Please select **all options** that apply

1. Under 5 years old
2. 5 to 7 years old
3. 8 to 10 years old
4. 11 to 12 years old
5. 13 to 15 years old
6. 16 or 17 years old

Q46

What is your highest level of education?

1. GCSEs/O levels
2. AS Levels
3. A-levels
4. Post Graduate Diploma
5. University Degree
6. Masters Degree
7. Doctorate
8. Welsh baccalaureate
9. International baccalaureate
10. Higher grade/advanced higher (Scotland)
11. Certificate of sixth year studies
12. Prefer not to say
13. Other (please specify) **[free text]**

Q47

What is your overall ANNUAL household income?

1. Up to £10,000
2. £10,001 - £20,000
3. £20,001 - £30,000
4. £30,001 - £40,000
5. £40,001 - £50,000
6. £50,001 - £60,000
7. £60,001 - £70,000
8. £70,001 - £80,000
9. £80,001 - £90,000
10. £90,001 - £100,000
11. More than £100,001
12. Prefer not to say

Q48

If you are happy to, please provide your full postcode (e.g., AL9 7TA) **[free text]**

DOG HEALTH QUESTIONS (PARTICIPANTS WHO STILL OWN THEIR DOG):

In this section we would like to understand any changes to your dog's access to veterinary care in the last 9 months. In addition, we would like to understand any changes surrounding your dog's health since the last survey you completed, for which you may or may not have sought veterinary advice.

Some of these questions are very similar to those asked in the previous surveys, but for some owners the answers may have changed.

Taking the time to answer these questions will greatly help our overall understanding of dog welfare and veterinary care in the UK and enable us to help improve the lives of dogs in the future.

Q49

Is your dog currently registered with a veterinary clinic(s)?

1. Yes **[SURVEY LOGIC – skip question 50]**
2. No **[SURVEY LOGIC – skip questions 51-57]**

Q50

Why is your dog not currently registered with a veterinary clinic(s)?

1. The clinic my dog was registered at removed us from their books
2. The clinic my dog was registered at closed down
3. I have not registered as yet, but I intend to in the future
4. I have not registered and do not intend to unless my dog becomes ill
5. I have tried but have been unable to find a practice that will register us
6. Other (please describe below**) [free text]**

Q51

Has your dog attended a veterinary clinic(s) or had a home visit from a veterinary professional for any **health problem(s)** in the last 9 months?

N.B. This does ***NOT*** *include attendances/visits for routine or preventative care (e.g., neutering, vaccination, microchipping, nail clipping, anal gland expression, routine check-ups, worming treatments, etc.).*

1. No **[SURVEY LOGIC – skip questions 52-54]**
2. I can't remember **[SURVEY LOGIC – skip questions 52-54]**
3. Yes (please give an approximate total number of visits for health problems in the last 9 months below as a whole number) **[number box]**

Q52

In your own words, please describe any **health problem(s)** and related treatments your dog has been to a veterinary clinic(s) for in the last 9 months. **[free text]**

N.B. This does ***NOT*** *include routine or preventative care (e.g., neutering, vaccination, microchipping, nail clipping, anal gland expression, routine check-ups, worming treatments, etc.).*

Q53

Does your dog have any ongoing **health problem(s)** that still require appointments/treatment/monitoring?

*N.B. This does **NOT** include routine or preventative care (e.g., neutering, vaccination, microchipping, nail clipping, anal gland expression, routine check-ups, worming treatments, etc.).*

1. No
2. I'm not sure
3. Yes (please describe below) **[free text]**

Q54

Please approximate the total cost (in £ GBP) of veterinary care for your dog's **health problem(s)** in the last 9 months or leave blank if you would prefer not to say. **[number box]**

*N.B. This should include any money that formed part of an insurance claim, if relevant, and any monies spent on prescription diets. This does **NOT** include routine or preventative care (e.g., neutering, vaccination, microchipping, nail clipping, anal gland expression, routine check-ups, worming treatments, etc.).*

Q55

How easy have you/your household found accessing veterinary care for your dog in the last 9 months?

Please select **all options** that apply.

N.B. This includes appointments which were online/via telephone as well as those in-person.

[Columns]:

a. Always easy
b. Sometimes easy
c. Rarely easy
d. Never easy
e. I haven't tried to make this type of appointment
f. I'm not sure/I can't remember
g. Not applicable

[Rows]:

1. Getting an appointment with my first-choice veterinary clinic
2. Getting an appointment with my preferred veterinary surgeon from my vet practice (if you have one)
3. Getting an appointment to see any veterinary surgeon
4. Getting an appointment to see a veterinary nurse
5. Getting an appointment with any veterinary clinic for an out of hours service (e.g., evenings, weekends)
6. Getting an appointment as quickly as I felt it was needed

Q56

How have you/other members of your household accessed veterinary care for your dog in the last 9 months?

Please select **all options** that apply.

[Columns]:

a. Always
b. Sometimes
c. Rarely
d. Never
e. I'm not sure/I can't remember

[Rows]:

1. Face-to-face consultations, accompanying my dog during their consultation
2. Consultations via video call with my usual veterinary practice or their out-of-hours provider (e.g., Zoom, Teams, FaceTime, WhatsApp, etc.)
3. Free consultations via my insurance company's remote video platform/telephone line
4. Paid for consultations via a remote veterinary service/telephone line
5. Telephone consultations with my usual veterinary practice or their out-of-hours provider

Q57

Please describe in your own words anything else (good or bad) that you would like to share about accessing veterinary care for your dog in the last 9 months. **[free text]**

Q58

Compared to my expectations when I first acquired my dog, the amount I have spent at veterinary clinic(s) on my dog's health in the last 9 months has been... **[free text]**

N.B. If you would like to explain your answer further in your own words, please feel free to do so below.

1. Less than I expected
2. As I expected
3. More than I expected
4. I'm not sure/I can't remember

Q59(a)

Has your dog shown any of the following in the last 9 months, regardless of whether you sought veterinary advice or not?

Please select **all options** that apply.

[Columns]:

a. No
b. Yes, but my dog did not need veterinary advice/to attend a veterinary appointment
c. Yes, and my dog did need veterinary advice/to attend a veterinary appointment
d. I'm not sure/I can't remember

[Rows]:

1. Runny and/or red eye(s)
2. Diarrhoea and/or runny faeces
3. Vomiting (being sick)
4. Worms in faeces

5. Fleas/other parasites visible in fur/on skin
6. Hair loss
7. Wounds/sore areas of skin
8. Frequent itching/licking
9. Coughing
10. Sore or infected ear(s)
11. Problems with their anal glands
12. Dental problems
13. Lameness
14. Overgrown nails
15. Being overweight
16. Losing weight
17. Arthritis
18. Lumps
19. Funny turns (suspected/confirmed to be fits/seizures)
20. Urinary incontinence (leaking urine)
21. Breathing problems
22. Eating non-food items (e.g., cloth, plastic, stones, faeces)
23. Other (please specify below) **[Q59(b)]**

Q59(b)

Please describe here in your own words any other health concerns your dog has had in the last 9 months that were not included in the list above. **[free text]**

N.B. Please also indicate whether veterinary advice was sought/your dog attended a veterinary appointment, or whether this was not necessary.

Q60

Is your dog insured?

N.B. We appreciate this is very similar to a previous question regarding the rising cost-of-living, but for some participants that previous question was not relevant.

1. Yes – and I intend to keep them insured
2. Yes – at the moment, but I'm undecided whether to continue with it at the next renewal
3. No – and I do not plan to insure them
4. No – but I plan to insure them in the future
5. No – I have cancelled or did not renew their policy
6. No – I have never heard of pet insurance
7. No – other (please specify below) **[free text]**

Q61

Which of the following routine vaccines has your dog received.

*N.B. Routine vaccinations do **NOT** include those for rabies or kennel cough.*

Please select **all options** that apply.

[Columns]:

a. No
b. Yes
c. I'm not sure/I can't remember

[Rows]:

1. Primary puppy vaccines at 8-16 weeks old
2. Year 1 booster (at 12-14 months)
3. Year 2 booster (at 24-26 months)
4. My dog has had/I plan for them to get a Year 3 booster (at 36-38 months)
5. I use blood testing (titre testing) to assess whether my dog needs to be vaccinated

Q62

Does your dog receive any regular flea and/or worming treatments and if so, where do you acquire these?

N.B. We appreciate this is very similar to a previous question regarding the rising cost-of-living, but for some participants that previous question was not relevant.

Please select **all options** that apply.

1. Yes – I acquire these through my vet or online WITH prescription
2. Yes – I acquire these from a shop or online WITHOUT prescription
3. Yes – I use natural/homeopathic remedies
4. No – I have chosen not to use any products regularly to prevent fleas and/or worms
5. I'm not sure

Q63

Has your dog been microchipped, and if so, are the details on record up to date?

1. Yes, and the details are for my name/current address
2. Yes, but I have not yet updated the details to include a change of name/address
3. Yes, but I'm not sure if my dogs microchip details are up to date
4. No, my dog has not been microchipped
5. I'm not sure if my dog has been microchipped

Q64

Has your dog been neutered (spayed/castrated)?

N.B. We appreciate this is very similar to a previous question regarding the rising cost-of-living, but for some participants that previous question was not relevant.

1. Yes **[SURVEY LOGIC – skip questions 65 and 66]**
2. No

Q65

Have you/do you intend to breed from your dog?

N.B. We appreciate this is very similar to a previous question regarding the rising cost-of-living, but for some participants that previous question was not relevant.

1. No, I do not plan to breed from them **[SURVEY LOGIC – skip question 66]**
2. I've not decided yet, but it could be something I may consider in the future **[SURVEY LOGIC – skip question 66]**
3. Yes, they will be bred from in the future, but have not been bred from yet **[SURVEY LOGIC – skip question 66]**
4. Yes, they have already been bred from

Q66
How many litters has your bitch/dog given birth to/fathered?
1. My bitch/dog is currently pregnant/has fathered a litter that has yet to be born (if applicable)
2. My bitch/dog has given birth to/fathered one litter of puppies
3. My bitch/dog has given birth to/fathered two litters of puppies
4. My bitch/dog has given birth to/fathered more than two litters of puppies

DOG BEHAVIOUR AND LIFESTYLE QUESTIONS (PARTICIPANTS WHO STILL OWN THEIR DOG):

In this section we would like to understand any changes surrounding your dog's behaviour and lifestyle in the last 9 months.

Once again, we understand that some of these questions are very similar to those asked in previous surveys, but for some owners the answers may have changed.

Taking the time to answer these questions will greatly help our overall understanding of dog welfare and enable us to help improve the lives of dogs in the future.

Q67
In the last 9 months, have you or someone in your household attended any training classes with your dog such as obedience, gundog training, scentwork, ringcraft, agility classes, etc.?
1. Yes
2. No

Q68
In the last 9 months, have you or anyone in your household used any of the following aids or methods on/with your dog, to try and change any aspect of their behaviour?
Please select **all options** that apply.
[Columns]:
a. Yes
b. No

[Rows]:
1. Allowing sniffing time/interaction with the environment
2. Anti-bark 'Husher' muzzle

3. Bark-activated citronella/vibration/ultrasonic collar
4. Choke/check chain
5. Clicker training
6. Electric collar
7. Electronic boundary fence
8. Food/treats
9. Harness
10. Head collar
11. Lead corrections (e.g., quickly yanking back on/jerking the lead if your dog pulls)
12. Pet Corrector™
13. Physical correction (e.g., smacking, tapping their nose, hitting, pinching)
14. Physical touch, e.g., stroking or patting
15. Physically moving your dog (e.g., pushing on your dog's hindquarters to get them into a sit, pushing them off furniture or if they jump up at you)
16. Playing with other dogs
17. Playing with you/another household member
18. Prong collar
19. Rattle bottle/cans/discs as a distractor
20. Rubbing their nose in faeces/urine if they toilet in an inappropriate location
21. Shouting at them or telling them off
22. "Time out": shutting your dog away within the home, away from people (e.g., in another room, in their crate)
23. "Time out": shutting your dog outdoors (e.g., in the garden/yard or an outside kennel)
24. Slip lead
25. Toys
26. Verbal praise
27. Water pistol/spray bottle

Q69

In the last 9 months, have you or anyone in your household used any other aids or methods not listed previously on/with your dog to try and change any aspect of their behaviour?

1. No
2. Yes (please describe below) **[free text]**

Q70

Compared to my expectations when I first acquired my dog, training and ongoing maintenance of their basic obedience in the last 9 months has been... **[free text]**

N.B. If you would like to explain your answer further in your own words, please feel free to do so below.

1. Harder than I expected
2. As I expected
3. Easier than I expected
4. I'm not sure/I can't remember

Q71(a)

In the last 9 months, have you considered that your dog has a problem with the following behaviours? If so, have you sought advice either formally or informally?

Please select **all advice options** that apply to each individual behaviour.

[Columns]:

a. No
b. Yes, but I did not seek advice
c. Yes, advice from a veterinary professional
d. Yes, advice from a dog trainer
e. Yes, advice from a dog behaviourist
f. Yes, advice from my dog's breeder
g. Yes, advice from another source (e.g., friend, family book, social media, etc.)
h. I'm not sure

[Rows]:

1. Pulling on their lead
2. Jumping up at people
3. Barking at other dogs
4. Not coming back when called
5. Fear of loud sounds (e.g., fireworks, thunderstorms)
6. Chasing (e.g., cats, wildlife, traffic)
7. Clinginess (e.g., following you, sitting close)
8. Anxiety/fear around other dogs
9. Anxiety/fear around people in your household (including you)
10. Anxiety/fear around unfamiliar people
11. Aggression towards other dogs
12. Aggression towards people in your household (including you)
13. Aggression towards unfamiliar people
14. Guarding of food, toys, or other items
15. Mouthing
16. Barking or howling
17. Being destructive
18. Toileting (weeing or pooing) indoors
19. Eating their or other dogs' faeces
20. Mounting/humping other dogs, people or objects
21. Other (please describe below) **[Q71(b)]**

Q71(b)

Please describe here in your own words, any other behaviours not listed above that your dog has had a problem with in the last 9 months, including where you sought advice if relevant. **[free text]**

Q72

In the last 9 months, has your dog performed any of the following behaviours?

Please select **all options** that apply.

[Columns]:

a. Yes
b. No
c. I'm not sure

[Rows]:

1. Repeatedly spins in a circle and/or chases their tail, i.e., turns in a circle more than three times, and may or may not catch their tail **[SURVEY LOGIC – send to question 73 if option (a) 'Yes' was selected]**
2. Chases, stalks, pounces, stares or bites at lights, reflections and/or shadows **[SURVEY LOGIC – send to question 74 if option (a) 'Yes' was selected]**
3. Stares into the sky and snaps/bites (WITHOUT flies/insects being there), which may be accompanied by jumping, licking, and swallowing **[SURVEY LOGIC – send to question 75 if option (a) 'Yes' was selected]**
4. Repeatedly seeks out and eats non-food items (e.g., cloth, plastic, stones, faeces) **[SURVEY LOGIC – send to question 76 if option (a) 'Yes' was selected]**

Q73

Have you sought any advice (formally or informally) about your dog's behaviour of repeatedly spinning in a circle and/or chasing their tail, i.e., turning in a circle more than three times, and maybe or maybe not catching their tail in the last 9 months?

Please select **all options** that apply.

1. Yes, from a veterinary professional
2. Yes, from a dog trainer
3. Yes, from a dog behaviourist
4. Yes, from my dog's breeder
5. Yes, advice from another source (e.g., friend, family, book, social media, etc.)
6. No
7. I can't remember

Q74

Have you sought any advice (formally or informally) about your dog's behaviour of chasing, stalking, pouncing, staring or biting at lights, reflections and/or shadows in the last 9 months?

Please select **all options** that apply.

1. Yes, from a veterinary professional
2. Yes, from a dog trainer
3. Yes, from a dog behaviourist
4. Yes, from my dog's breeder
5. Yes, advice from another source (e.g., friend, family, book, social media, etc.)
6. No

7. I can't remember

Q75

Have you sought any advice (formally or informally) about your dog's behaviour of staring into the sky and snapping/biting (WITHOUT flies/insects being there), which may be accompanied by jumping, licking, and swallowing in the last 9 months?

Please select **all options** that apply.

1. Yes, from a veterinary professional
2. Yes, from a dog trainer
3. Yes, from a dog behaviourist
4. Yes, from my dog's breeder
5. Yes, advice from another source (e.g., friend, family, book, social media, etc.)
6. No
7. I can't remember

Q76

Have you sought any advice (formally or informally) about your dog's behaviour of repeatedly seeking out and eating non-food items (e.g., cloth, plastic, stones, faeces) in the last 9 months?

Please select **all options** that apply.

1. Yes, from a veterinary professional
2. Yes, from a dog trainer
3. Yes, from a dog behaviourist
4. Yes, from my dog's breeder
5. Yes, advice from another source (e.g., friend, family, book, social media, etc.)
6. No
7. I can't remember

Q77(a)

Where have you sought information about any aspect of your dog's general behaviour or training in the last 9 months?

Please select **all options** that apply.

[Columns]:

a. Yes
b. No
c. I can't remember

[Rows]:

1. I have not needed to seek any information about my dog's general behaviour or training in the last 9 months
2. A veterinary professional (e.g., veterinary surgeon, veterinary nurse)
3. Dog trainer
4. Dog behaviourist
5. My dog's breeder

6. Friends or family who own or have owned a dog
7. The Kennel Club website
8. An animal charity website, e.g., Dogs Trust, RSPCA, PDSA, etc.
9. A breed/crossbreed-specific online resource (e.g., website/forum)
10. Social media sites, e.g., Facebook, Instagram
11. YouTube
12. Book(s)
13. TV programmes
14. Dog-specific magazine(s)
15. My own experience from owning dogs in the past

Q77(b)

Please describe here in your own words, any other sources of information not listed above that you have used for any aspect of your dog's general behaviour and training in the last 9 months. **[free text]**

Q78

Compared to my expectations when I first acquired my dog, their behaviour in the last 9 months has been... **[free text]**

N.B. If you would like to explain your answer further in your own words, please feel free to do so below.

1. Worse than I expected
2. As I expected
3. Better than I expected
4. I'm not sure/I can't remember

Q79

What length of time is your dog is currently left at home alone (without any people present in the home) on a typical weekday?

N.B. This should be the longest period of time that your dog is left without a break/visit, e.g., if you leave your home between 8 am to 5 pm but either yourself or someone else visits your dog at 1 pm, the longest stretch they are left alone is 5 hours.

1. Never left alone **[SURVEY LOGIC – skip question 80 and 82]**
2. Under 1 hour
3. Up to 2 hours
4. Up to 3 hours
5. Up to 4 hours
6. Up to 5 hours
7. Up to 6 hours
8. Up to 7 hours
9. Up to 8 hours
10. Up to 9 hours
11. Up to 10 hours
12. Over 10 hours

Q80
When your dog is left at home alone (without any people present in the home), which of the following spaces do they most often have access to?

1. A crate/indoor kennel (with the door closed)
2. Loose in a single room, e.g., kitchen
3. Loose in multiple rooms, e.g., hallway, living room, kitchen
4. Outdoor kennel (with the door closed)
5. Outdoor kennel (with the door open)
6. Loose in an enclosed outdoor space, e.g., garden, yard
7. Other (please describe below) **[free text]**

Q81(a)
In the last 9 months has your dog shown any of the following behaviours when at home with you/other household members while you are relaxing (e.g., watching TV)?
Please select **all options** that apply.
[Columns]:

a. Yes
b. No

[Rows]:

1. Destructive behaviour (e.g., chewing and causing damage to household items such as furniture, not including their own toys/treats)
2. Scratches doors or around doors
3. Barks
4. Howls
5. Whines/cries
6. Wees in an inappropriate place, e.g., inside the home if usually housetrained
7. Poos in an inappropriate place, e.g., inside the home if usually housetrained
8. Spins (repeatedly turns in a circle)
9. Paces (walks repetitively around the home/room, unable to settle)

Q81(b)
Please describe here in your own words, any other behaviours not listed above that your dog has shown in the last 9 months when at home with you/other household members while you are relaxing (e.g., watching TV)? **[free text]**

Q82
To your knowledge (e.g., from seeing evidence on your return home, on a video camera, being told by neighbours, etc.), has your dog shown any of the following behaviours when left at home alone (without any people present in the home) in the last 9 months?
Please select **all options** that apply.
[Columns]:

a. Yes
b. I suspect they might
c. No
d. I don't know

[Rows]:

1. Destructive behaviour (e.g., chewing and causing damage to household items such as furniture, not including their own toys/treats) **[SURVEY LOGIC – send to question 84 if option (a) 'Yes' was selected]**
2. Scratches doors or around doors **[SURVEY LOGIC – send to question 84 if option (a) 'Yes' was selected]**
3. Barks **[SURVEY LOGIC – send to question 84 if option (a) 'Yes' was selected]**
4. Howls **[SURVEY LOGIC – send to question 84 if option (a) 'Yes' was selected]**
5. Whines/cries **[SURVEY LOGIC – send to question 84 if option (a) 'Yes' was selected]**
6. Wees in an inappropriate place, e.g., inside the home if usually housetrained **[SURVEY LOGIC – send to question 84 if option (a) 'Yes' was selected]**
7. Poos in an inappropriate place, e.g., inside the home if usually housetrained **[SURVEY LOGIC – send to question 84 if option (a) 'Yes' was selected]**
8. Spins (repeatedly turns in a circle) **[SURVEY LOGIC – send to question 84 if option (a) 'Yes' was selected]**
9. Paces (walks repetitively around the home/room, unable to settle) **[SURVEY LOGIC – send to question 84 if option (a) 'Yes' was selected]**

Q83

To your knowledge (e.g., from seeing evidence on your return home, on a video camera, being told by neighbours, etc.), has your dog shown any other behaviours not listed above when left at home alone (without any people present in the home) in the last 9 months?

1. No **[SURVEY LOGIC – skip question 84]**
2. Yes (please describe below in your own words) **[free text]**

Q84

When your dog has previously shown any of these behaviours when left at home alone, how have you responded soon after your return?
Please select **all options** that apply.

1. No reaction, e.g., ignored what has happened
2. Stroked/patted/cuddled them
3. Played with them
4. Told them off (e.g., shouted at them, said no)
5. Physically reprimanded them (e.g., tapped them, hit them, held them down)
6. Put them in a 'time out' (e.g., on their own in a restricted indoor space, or outside)
7. Other (please describe below) **[free text]**

DOG ROUTINE AND CARE QUESTIONS (PARTICIPANTS WHO STILL OWN THEIR DOG):

In this section we would like to understand a little bit about your dog's routine and care.

Q85

Where do you currently work?

1. I work from home **[SURVEY LOGIC – skip questions 86 and 87]**
2. I go out to work
3. I work from home AND go out to work
4. N/A – I am unemployed **[SURVEY LOGIC – skip questions 86 and 87]**
5. N/A – I am retired **[SURVEY LOGIC – skip questions 86 and 87]**
6. N/A – I am unable to work (e.g., due to ill health, etc.) **[SURVEY LOGIC – skip questions 86 and 87]**

Q86

Do you take your dog to your place of work?

1. Yes, always **[SURVEY LOGIC – skip question 87]**
2. Yes, most times (at least half or more of my workdays) **[SURVEY LOGIC – skip question 87]**
3. Yes, occasionally (less than half of my workdays) **[SURVEY LOGIC – skip question 87]**
4. No

Q87

Why do you not take your dog to your place of work?
Please select **all options** that apply.

1. I don't want to
2. My workplace is not suitable for dogs
3. My workplace doesn't allow it
4. I have tried taking my dog to work previously but their behaviour was not suited to my work environment/they found it too stressful
5. I have not asked but may request to do this in the future
6. Another member of my household takes my dog to work with them
7. Other reasons (please describe below) **[free text]**

Q88

Has there been a change, for any reason, in how long your dog is left at home alone without any people present over the last 9 months?
N.B. We appreciate this is very similar to a previous question regarding the rising cost-of-living, but for some participants that previous question was not relevant.

1. No, left alone for the same amount of time **[SURVEY LOGIC – skip question 89]**
2. Yes, left alone for less time than before
3. Yes, left for more time than before (under 4 hours in total at a time)
4. Yes, left for more time than before (over 4 hours in total at a time)

Q89

Over the last 9 months, there been a change in how long my dog is left at home alone without any people present because…

N.B. Household members include yourself.

1. Household members who used to go out to work are now retired
2. Household members who used to go out to work/study are now unemployed
3. Additional household members now live with me/us and care for the dog
4. Household members who used to be at home all the time now go out to work/study
5. Household members now go out to work/study for longer
6. Household members who used to be at home all the time no longer live with me/us
7. Other (please describe below) **[free text]**

Q90

Who is involved in your dog's day-to-day care other than your immediate household?

Please select **all options** that apply.

1. Doggy daycare
2. Dog walker (professional)
3. Family member(s) I do not live with
4. Friend(s)
5. Dog sharing services (e.g., BorrowMyDoggy)
6. None of the above
7. Other (please describe below) **[free text]**

Q91

How often is your dog walked on average (this includes walks by you/members of your household and others, e.g., friends, family, professional dog walkers)?

1. More than twice per day
2. Twice per day
3. Once per day
4. Several times a week
5. Once a week
6. Less than once a week
7. Never

Q92

How often on average is your dog exercised off lead (if ever)?

1. More than twice per day
2. Twice per day
3. Once per day
4. Several times a week
5. Once a week
6. Less than once a week

7. Never

Q93

Compared to my expectations when I first acquired my dog, their exercise requirements in the last 9 months have been... **[free text]**

N.B. If you would like to explain your answer further in your own words, please feel free to do so below.

1. Less than I expected
2. As I expected
3. More than I expected
4. I'm not sure/I can't remember

Q94

What diet is your dog typically fed at the moment?

Please select **all options** that apply.

1. Complete dry (e.g., kibble)
2. Complete wet (e.g., tinned food, pouches)
3. Mixer biscuits
4. Commercially available raw dog food
5. Raw diet that I prepare myself (e.g., buying own meat from butchers/shops)
6. Left over human food
7. Dog treats
8. Home cooked diet
9. Vegetarian diet
10. Vegan diet
11. Insect protein-based diet
12. Prescription diet, used under veterinary direction

Q95

Compared to my expectations when I first acquired my dog, their maintenance levels (e.g., daily time dedicated to your dog's upkeep such as feeding, training but NOT exercise or grooming) in the last 9 months have been... **[free text]**

N.B. If you would like to explain your answer further in your own words, please feel free to do so below.

1. Less than I expected
2. As I expected
3. More than I expected
4. I'm not sure/I can't remember

Q96

Compared to my expectations when I first acquired my dog, the time involved in grooming them in the last 9 months have been... **[free text]**

N.B. If you would like to explain your answer further in your own words, please feel free to do so below.

1. Less than I expected

2. As I expected
3. More than I expected
4. I’m not sure/I can’t remember

LIVING WITH THEIR DOG QUESTIONS (PARTICIPANTS WHO STILL OWN THEIR DOG):

In this section we would like to understand how living with your dog has met, or not, any expectations you may have had before or soon after you purchased them.

Q97

Compared to when I first acquired my dog, the relationship I have had with them in the last 9 months has been... **[free text]**

N.B. If you would like to explain your answer further in your own words, please feel free to do so below.

1. Worse than I expected
2. As I expected
3. Better than I expected
4. I’m not sure/I can’t remember

Q98

Please consider the following questions and report approximately how often you and your dog take part in the activities (if ever).

How often do you…

[Columns]:

a. At least once a day
b. Once every few days
c. Once a week
d. Once a month
e. Never

[Rows]:

1. Play games with your dog?
2. Take your dog to visit people?
3. Give your dog food treats?
4. Kiss your dog?
5. Take your dog in the car?
6. Hug your dog?
7. Have your dog with you while relaxing, e.g., watching TV?
8. Groom your dog?

Q99

Please consider the following questions and report approximately how often (if ever) they apply.

How often…

[Columns]:

a. Once a day
b. Once a week
c. Once a month
d. Once a year
e. Never

[Rows]:

1. Do you tell your dog things you don't tell anyone else?
2. Do you feel that looking after your dog is a chore?
3. Does your dog stop you doing things you want to?
4. Do you feel that having a dog is more trouble than it is worth?

Q100

Please consider the following question and report approximately how often (if ever) it applies.
How often do you…

[Columns]:

a. Once a week
b. Once a fortnight
c. Once a month
d. A couple of times a year
e. Never

[Rows]:

1. Buy your dog presents?

Q101

Please consider the following statements and indicate your level of agreement with them, from strongly agreeing, through to strongly disagreeing.

[Columns]:

a. Strongly agree
b. Agree
c. Neither agree nor disagree
d. Disagree
e. Strongly disagree

[Rows]:

1. My dog helps me get through tough times
2. My dog is there whenever I need to be comforted
3. I would like to have my dog near me all the time
4. My dog provides me with constant companionship
5. If everyone else left me, my dog would still be there for me
6. My dog gives me a reason to get up in the morning
7. I wish my dog and I never had to be apart
8. My dog is constantly attentive to me
9. It is annoying that I sometimes have to change my plans because of my dog

10. It bothers me that my dog stops me doing things I enjoyed doing before I owned it
11. There are major aspects of owning a dog I don't like
12. My dog makes too much mess
13. My dog costs too much money

Q102
Please consider the following question and select the choice that best matches your answer.
[Columns]:

a. Very traumatic
b. Traumatic
c. Neither traumatic nor untraumatic
d. Untraumatic
e. Very untraumatic

[Rows]:

1. How traumatic do you think it will be for you when your dog dies?

Q103
Please consider the following question and select the choice that best matches your answer.
[Columns]:

a. Very hard
b. Hard
c. Neither hard nor easy
d. Easy
e. Very easy

[Rows]:

1. How hard it is to look after your dog?

Q104
Have you considered rehoming your dog in the last 9 months?

1. No – I have never considered rehoming them **[SURVEY LOGIC – skip questions 105 and 106]**
2. Yes – but I am no longer considering rehoming them
3. Yes – and I am still considering rehoming my dog

Q105
If you are comfortable doing so, please describe in your own words why you considered rehoming your dog. **[free text]**

Q106
If you are comfortable doing so, please describe in your own words what you think would help/have helped you to avoid having to consider rehoming your dog. This could be advice pre- or post-purchase, access to resources, etc. **[free text]**

DEBRIEF SECTION 1 (PARTICIPANTS WHO STILL OWN THEIR DOG):

Thank you very much for taking part in our study, your time and effort in completing these surveys is very much appreciated.

We understand that this survey may have worried some owners who have faced challenges with dog ownership. We have therefore collated some information from some reputable sources below.

Worried about your dog's health?

If you have any concerns about your dog's health you should contact your vet in the first instance who can examine your dog, take a thorough history and suggest further investigations or treatments that may be necessary. Register with a local vet if you have not yet done so – to find a local veterinary surgeon, please visit the following website:

https://findavet.rcvs.org.uk/home/

If you are struggling with the rising cost-of-living, you may meet the eligibility criteria for subsidised/free veterinary services available from charities including the PDSA, RSPCA and Blue Cross:

https://www.pdsa.org.uk/what-we-do/treat
https://www.rspca.org.uk/whatwedo/care/vetcare
https://www.bluecross.org.uk/veterinary

Worried about your dog's behaviour?

Lockdown has been a challenging time for the mental wellbeing of dogs, with many households experiencing dramatic changes in their routines, which may put dogs at risk of separation anxiety. In addition, restrictions from lockdown meant some puppies missed out on vital experiences in their early life which may have left them vulnerable to behaviour problems in the future.
If you are worried about a behavioural problem your dog has developed, talk to your vet about being referred to an accredited animal or veterinary behaviourist via the Animal Behaviour and Training Council (ABTC):

https://abtc.org.uk/practitioners/

The ABTC also have links to accredited dog trainers across the UK and dog training resources on their website:

https://abtc.org.uk/owners/

DEBRIEF SECTION 2 (ALL PARTICIPANTS):

Thank you for completing this survey. We would like to thank you for the time and support you have so generously given to this research study since 2020.

The COVID-19 pandemic and rising cost-of-living has created challenges for mental health across the UK. If you would like support or advice regarding your own mental health, the following websites may be useful:

- https://www.nhs.uk/oneyou/every-mind-matters/
- https://www.samaritans.org
