# Supplementary material for: Impacts of the ‘Cost-of-Living Crisis’ on UK-Based Owners of Pandemic Puppies
Source: Animals (Basel). 2026 Aug 24;16(17):2656. doi: 10.3390/ani16172656 (PMC13564328; doi:10.3390/ani16172656)
Supplement: Supplementary file 1 [file animals-16-02656-s001.zip › File S2.pdf]

TABLE S1. Variables used for analysis.

Variables used for univariable* analysis. ‡Categorical variable, binary. §Categorical variable, more than two categories. ¶Continuous variable.

	Variable	Survey derived from
**Owner/household demographics**	‡First-time dog owner in 2020	2020
	§Owner age in 2020	2020
	§Owner gender	2020
	§Annual household income	COLC
	§Owners current employment status	36 month longitudinal
	§Owners educational level	COLC
	‡Owner lives alone/only adult in the household	36 month longitudinal
	‡Child ≤18 years in household	36 month longitudinal
	‡Another dog(s) in household	2020, updated 36 month longitudinal
	§UK region of residence	36 month longitudinal
**Dog demographics**	‡Dog sex	2020
	‡Neuter status	2020, updated 36 month longitudinal
	§Breed/crossbreed categorisation	2020
	§Poodle-type categorisation	2020
	§Top 12 most common breeds at 36 months	2020, analysed at 36 months
	§UK Royal Kennel Club breed group	2020
	§Typical adult bodyweight categorisation	2020
**Purchase motivations for breed/crossbreed**	‡Affordable cost of upkeep	2020
	‡Generally healthy breed	2020
	‡Easy to train	2020
**Owner expectations**	‡Veterinary costs	36 month longitudinal
	‡Training	36 month longitudinal
	‡Behaviour	36 month longitudinal
	‡Maintenance	36 month longitudinal
	‡Grooming	36 month longitudinal
	‡Exercise	36 month longitudinal
**Dog health**	‡Attended a veterinary clinic for a health problem in the preceding 9 months	36 month longitudinal
	¶Number of visits to a veterinary clinic for a health problem in the preceding 9 months	36 month longitudinal
	¶Number of health disorder groups requiring visits to a veterinary clinic in the preceding 9 months	36 month longitudinal
	‡Ongoing health issues requiring veterinary visits	36 month longitudinal
	¶Total number of ongoing health disorder groups requiring veterinary visits	36 month longitudinal
	¶Total spend on health disorders at a veterinary clinic in the preceding 9 months	36 month longitudinal
	¶Total number of health disorder groups in the preceding 9 months, whether or not veterinary advice sought	36 month longitudinal
	§Worming	36 month longitudinal
	‡Vaccinations up to date	36 month longitudinal
	‡Insurance status	2020, updated 36 month longitudinal

TABLE S1. Contd…

	Variable	Survey derived from
**Dog behaviour**	¶Total number of 'problem behaviours' in the preceding 9 months	36 month longitudinal
	‡Displayed a 'control behaviour' in the preceding 9 months	36 month longitudinal
	‡ Displayed an 'attention-seeking behaviour' in the preceding 9 months	36 month longitudinal
	‡Displayed an 'aggressive behaviour' in the preceding 9 months	36 month longitudinal
	‡Displayed a 'fear/avoidance behaviour' in the preceding 9 months	36 month longitudinal
	‡Displayed a 'reaction to familiar people behaviour' in the preceding 9 months	36 month longitudinal
	‡Displayed a 'reaction to unfamiliar people behaviour' in the preceding 9 months	36 month longitudinal
	‡Displayed a 'reaction to dogs behaviour' in the preceding 9 months	36 month longitudinal
**Cost-of-living impacts, dog health**	‡Change in decision to seek advice for dog health issues from a veterinary surgeon in the preceding 9 months	COLC
	§Change in decision to insure dog	COLC
	§Change in decision to vaccinate dog	COLC
	§Change in decision to use preventative worming treatments	COLC
	§Change in decision to use preventative flea treatments	COLC
	§Change in decision to neuter dog	COLC
**Cost-of-living impacts, dog training and behaviour**	‡Change in decision to seek professional advice for dog training	COLC
	‡Change in decision on where to seek advice for dog behaviour issues in the preceding 9 months	COLC
**Cost-of-living impacts, dog maintenance**	‡Change in dogs diet in the preceding 9 months	COLC
	§Change in decision to use professional grooming	COLC
	§Change in decision to use paid extra help for day-today care	COLC
	§Change in decision to leave dog whilst going on holidays	COLC
	§Change in how long/often dog is left alone	COLC
**Cost-of-living impacts, household finances and negative canine welfare**	§Change in household finances in preceding 9 months	COLC
	§Change in household circumstances in next 6 months	COLC
	‡Negative impact on dog care currently	COLC
	‡Negative impact on dog care in the next 6 months	COLC
	‡Owning a dog is causing a negative impact on household lifestyle currently	COLC
	‡Owning a dog will cause a negative impact on household lifestyle in the next 6 months	COLC

TABLE S2. Consensus categorisation of potential welfare impact upon dogs resulting from owner-reported changes to dog healthcare and behaviour advice-seeking as a result of the Cost-of-Living Crisis (COLC), based upon multiple-choice question (MCQ) responses. Ambiguous changes could be interpreted as positive or negative for canine welfare depending upon respondents' individual circumstances and were therefore excluded from univariable analysis. * Multiple response options could be selected.

Question	Response Option	Potential Impact on Canine Welfare	Literature sources used to help assign classification
* Given that your dog had a health problem in the previous 9 months, has the rising cost-of-living affected your decisions and actions regarding seeking advice for your dog's health from a veterinary surgeon in the last 9 months?	Yes, I now qualify for charity veterinary care (e.g. PDSA, Blue Cross)	Potentially positive	[55-58]
	Yes, I've delayed seeking advice to see if a health issue got better by itself	Potentially negative	
	Yes, I've spent more time finding information about my dog's health issues online than I would otherwise have done so	Potentially negative	
	Yes, I've tried home remedies/suggestions from non-vets first before going to the vets	Potentially negative	
	Yes, I've not yet taken my dog for a minor problem (e.g. a new lump), whereas in the past I would have already done so	Potentially negative	
	Yes, I've cancelled/delayed a planned visit, investigation, operation or dental	Potentially negative	
	Yes, I've started using free online consultations linked to my pet insurance instead of/as well as going to the vets	Ambiguous	
	Yes, I've changed to a cheaper veterinary practice/shopped around on price	Ambiguous	
	No	None	
	No, not yet but I think it will in the future	None	

4. Muldoon, J.C.; Williams, J.M. When Having a Pet Becomes a Luxury You Can No Longer Afford. *Anthrozoos* **2024**, doi:10.1080/08927936.2024.2351276.

55. Bergh, A.; Lund, I.; Boström, A.; Hyytiäinen, H.; Asplund, K. A Systematic Review of Complementary and Alternative Veterinary Medicine: "Miscellaneous Therapies." *Animals* **2021**, *11*, 3356, doi:10.3390/ani11123356.

56. Muldoon, J.C.; Williams, J.M. Why Are Some People in the UK Reluctant to Seek Support for Their Pets? *Animal Welfare* **2024**, doi:10.1017/awf.2024.19.

57. Mellor, D.J.; Beausoleil, N.J.; Littlewood, K.E.; McLean, A.N.; McGreevy, P.D.; Jones, B.; Wilkins, C. The 2020 Five Domains Model: Including Human-Animal Interactions in Assessments of Animal Welfare. *Animals* **2020**, *10*, doi:10.3390/ani10101870.

TABLE S2. Contd…

Question	Response Option	Potential Impact on Canine Welfare	Literature sources used to help assign classification
Has/will the rising cost-of-living affect decisions regarding insuring your dog?	Yes, I have increased/will increase my insurance level of cover	Potentially positive	[55,59,60]
	Yes, I never insured my dog in the past, but have/will do so now	Potentially positive	
	Yes, I have reduced/will reduce my level of cover to make it cheaper	Potentially negative	
	Yes, I have cancelled/will cancel their insurance	Potentially negative	
	Yes, I have not/plan not to renew their insurance	Potentially negative	
	Yes, I have switched/will switch companies to a lower premium/higher excess	Potentially negative	
	No	None	

4. Muldoon, J.C.; Williams, J.M. When Having a Pet Becomes a Luxury You Can No Longer Afford. *Anthrozoos* **2024**, doi:10.1080/08927936.2024.2351276.

58. Becker, M.; Volk, H.A.; Kunzmann, P. Is Pet Health Insurance Able to Improve Veterinary Care? Why Pet Health Insurance for Dogs and Cats Has Limits: An Ethical Consideration on Pet Health Insurance. *Animals* **2022**, *12*, 1728–1728, doi:10.3390/ani12131728.

59. Williams, A.; Williams, B.; Hansen, C.R.; Coble, K.H. The Impact of Pet Health Insurance on Dog Owners' Spending for Veterinary Services. *Animals* **2020**, *10*, doi:10.3390/ani10071162.

TABLE S2. Contd…

Question	Response Option	Potential Impact on Canine Welfare	Literature sources used to help assign classification
Has/will the rising cost-of-living affect decisions regarding vaccinating your dog?	Yes, I never vaccinated my dog in the past but do/will do so now	Potentially positive	[61]
	Yes, my dog's vaccines are not up to date, but I will now ensure they are	Potentially positive	
	Yes, I vaccinated my dog in the past, but will no longer do so	Potentially negative	
	Yes, I usually titre test but will now just vaccinate	Ambiguous	
	No	None	
Has/will the rising cost-of-living affect your decisions regarding the brand, source or use of regular worm treatments for your dog?	Yes, I have started/will now start worming my dog	Potentially positive	[62-64]
	Yes, I have started to/plan to subscribe to a pet health club to pay for worm treatments via my vets	Potentially positive	
	Yes, I have not wormed/will no longer worm my dog	Potentially negative	
	Yes, I used to subscribe to a pet health club to pay for worm treatments via my vets but have stopped/plan to stop this	Potentially negative	
	Yes, I do/will worm my dog less often	Potentially negative	
	Yes, I have bought/will now buy wormer from somewhere other than the vets (e.g. to a cheaper source)	Ambiguous	
	No	None	

60. World Small Animal Veterinary; Association *2024 Guidelines for the Vaccination of Dogs and Cats – Compiled by the Vaccination Guidelines Group (VGG) of the World Small Animal Veterinary Association (WSAVA)*; **2024**, https://wsava.org/wp-content/uploads/2024/04/WSAVA-Vaccination-guidelines-2024.pdf, Accessed 26/07/2026.
61. Pennelegion, C.; Drake, J.; Wiseman, S.; Wright, I. Survey of UK Pet Owners Quantifying Internal Parasite Infection Risk and Deworming Recommendation Implications. *Parasit Vectors* **2020**, *13*, 218, doi:10.1186/s13071-020-04086-2.
62. Veterinary Voices UK *Fake News: Rabbit Ears Are Not Natural Dewormers* **2023**, https://www.vetvoices.co.uk/post/rabbit-ear-not-dewormer, Accessed 26/07/2026.
63. Hickey, K.; Smith, D.; Shields, E. *Qualitative Research on Pet Owners' Experiences of Buying Veterinary Services in the UK*; **2024**, https://assets.publishing.service.gov.uk/media/65eedd9d62ff4898bf87b261/Qualitative_Research_on_Pet_Owners__Experiences_of_Buying_Veterinary_Services_in_the_UK.pdf, Accessed 26/07/2026.

TABLE S2. Contd…

Question	Response Option	Potential Impact on Canine Welfare	Literature sources used to help assign classification
Has/will the rising cost-of-living affect your decisions regarding the brand, source or use of regular flea treatments for your dog?	Yes, I have started/will now start flea treating my dog	Potentially positive	[64-66]
	Yes, I have started to/plan to subscribe to a pet health club to pay for flea treatments via my vets	Potentially positive	
	Yes, I have not/will no longer flea treat my dog	Potentially negative	
	Yes, I used to subscribe to a pet health club to pay for flea treatments via my vets but have stopped/plan to stop this	Potentially negative	
	Yes, I do/will flea treat my dog less often	Potentially negative	
	Yes, I have bought/will now buy flea treatments from somewhere other than the vets (e.g. to a cheaper source)	Ambiguous	
	No	None	

63. Hickey, K.; Smith, D.; Shields, E. *Qualitative Research on Pet Owners' Experiences of Buying Veterinary Services in the UK*; **2024**, https://assets.publishing.service.gov.uk/media/65eedd9d62ff4898bf87b261/Qualitative_Research_on_Pet_Owners__Experiences_of_Buying_Veterinary_Services_in_the_UK.pdf, Accessed 26/07/2026.

64. Wong, S.; Elsheikha, H.; Dryden, M. Flea Product Efficacy, Pet Owners' Adherence and Treatment Failure: What's the Connection? *UK-VET Companion Animal* **2021**, *26*.

65. Farrell, S.; McGarry, J.; Noble, P.-J.M.; Pinchbeck, G.J.; Cantwell, S.; Radford, A.D.; Singleton, D.A. Seasonality and Other Risk Factors for Fleas Infestations in Domestic Dogs and Cats. *Med Vet Entomol* **2023**, *37*, 359–370, doi:10.1111/mve.12636.

TABLE S2. Contd…

Question	Response Option	Potential Impact on Canine Welfare	Literature sources used to help assign classification
Has/will the rising cost-of-living affect your decisions regarding neutering your dog?	N/A – my dog is already neutered	Ambiguous	[55,67]
	Yes, I have now had my dog neutered	Ambiguous	
	Yes, I now plan to neuter my dog	Ambiguous	
	Yes, I have cancelled plans to neuter my dog, but don't plan to breed from them	Ambiguous	
	Yes, I have cancelled plans to neuter my dog, and now plan to breed from them	Ambiguous	
	I'm not sure	Ambiguous	
	No, it has not affected my decision	None	
Given that your dog is not neutered, has/will the rising cost-of-living affect your decisions regarding breeding your dog?	Yes, I wasn't planning to breed from my dog, but now plan to breed from them	Ambiguous	[55,67]
	Yes, I was planning to breed from my dog, but now plan not to breed from them		
	No, it has not affected my decision	None	

4. Muldoon, J.C.; Williams, J.M. When Having a Pet Becomes a Luxury You Can No Longer Afford. *Anthrozoos* **2024**, doi:10.1080/08927936.2024.2351276.

66. RCVS Knowledge *Benefits and Risks of Neutering Pets – What Is the Evidence*; **2026**, https://www.rcvsknowledge.org/resource/benefits-and-risks-of-neutering-pets-what-is-the-evidence/#about-evidence-collections, Accessed 26/07/2026.

TABLE S2. Contd…

Question	Response Option	Potential Impact on Canine Welfare	Literature sources used to help assign classification
* Has the rising cost-of-living affected whether or how you access advice regarding any specific behaviour problems you have experienced with your dog in the last 9 months? *N.B. Behaviour professional refers to a qualified dog behaviourist or veterinary nurse/veterinary surgeon.*	Yes, I've used charity behaviour helplines (e.g. Dogs Trust)	Potentially positive	[68-70]
	Yes, I've delayed seeking advice to see if a behaviour problem got better by itself	Potentially negative	
	Yes, I've not sought advice from a behaviour professional, whereas in the past I would have done so	Potentially negative	
	Yes, I've started using free online consultations linked to my pet insurance instead of/before seeking referral to a behaviour professional in person	Potentially negative	
	Yes, I've cancelled a planned visit to see a behaviour professional regarding my dog's behaviour problem(s)	Potentially negative	
	Yes, I've tried suggestions from non-professionals (e.g. friends/family) instead of/before seeking referral to a behaviour professional	Potentially negative	
	Yes, I've tried suggestions from a dog trainer instead of/before seeking referral to a behaviour professional	Potentially negative	
	Yes, I've spent more time finding information about my dog's behaviour problems online than I would otherwise have done so	Potentially negative	
	Yes, I've changed to a cheaper behaviour professional/shopped around on price	Potentially negative	
	I'm not sure	Ambiguous	
	N/A – my dog has not had any behavioural problems in this time period	None	
	No	None	
	No, not yet but I think it will in the future	None	

67. Powdrill-Wells, N.; Taylor, S.; Melfi, V. Reducing Dog Relinquishment to Rescue Centres Due to Behaviour Problems: Identifying Cases to Target with an Advice Intervention at the Point of Relinquishment Request. *Animal* 2021, *11*, 2766, doi:10.3390/ani11102766.

Literature cited continued on next page…

TABLE S2. Contd…

Question	Response Option	Potential Impact on Canine Welfare	Literature sources used to help assign classification
* Has/will the rising cost-of-living affect any aspect of your dog's training, for example, attending training classes? *N.B. Behaviour professional refers to a qualified dog behaviourist or veterinary nurse/veterinary surgeon.*	Yes, I have not/will not purchase or have cancelled/plan to cancel training classes (in person/online) that I had previously wanted to attend	Potentially negative	[69,71]
	Yes, I have been/will be less likely seek professional advice for training issues	Potentially negative	
	N/A – I don't attend any training classes with my dog	None	
	No, it has not affected any training class attendance plans	None	
	No, I still prefer to seek professional advice when it comes to any training issues	None	

68. Barcelos, A.M.; Kargas, N.; Mills, D. The Effects of Dog Behavioural Problems on Owner Well-Being: A Review of the Literature and Future Directions. *International Symposium on Privacy Enhancing Technologies* **2024**, doi:10.3390/pets1010007

69. Malkani, R.; Paramasivam, S.; Wolfensohn, S. A Multidimensional Evaluation of the Factors in the Animal Welfare Assessment Grid (AWAG) That Are Associated with, and Predictive of, Behaviour Disorders in Dogs. *Animals* **2024**, *14*, doi:10.3390/ani14040528.

70. Puurunen, J.; Hakanen, E.; Salonen, M.; Mikkola, S.; Sulkama, S.; Araujo, C.L.; Lohi, H. Inadequate Socialisation, Inactivity, and Urban Living Environment Are Associated with Social Fearfulness in Pet Dogs. *Scientific Reports* **2020**, *10*, 3527–3527, doi:10.1038/s41598-020-60546-w.

TABLE S2. Contd…

Question	Response Option	Potential Impact on Canine Welfare	Literature sources used to help assign classification
* In the last 9 months, has there been a change in the diet you typically feed your dog, including the type, source or brand, that is due in part or entirely to the rising cost-of-living?	Yes, a change to a cheaper brand	Potentially negative	[72-74]
	Yes, a change from one general type of diet to another cheaper type of diet (e.g. raw diet to a complete dry diet, a prescription diet to non-prescription diet)	Potentially negative	
	Yes, a change in where I purchase my dog's food, to a cheaper source (e.g. from in store to online, from your vets to an online company, or to a different online source)	Ambiguous	
	Yes, a change but not related to the rising cost-of-living	None	
	No change	None	
Has/will the rising cost-of-living affect how often or whether your dog is professionally groomed?	Yes, my dog is being/will be professionally groomed more often	Potentially positive	[75-77]
	Yes, my dog is being/will be professionally groomed less often	Potentially negative	
	N/A – my dog has never been professionally groomed	None	
	No change	None	

71. Jobe, M.T.; Downs, K.M. Systematic Review and Meta-Analysis of Quality Claims Associated with Fresh Pet Food: Evaluating Scientific Evidence for Additives, Ingredient Quality, and Effects of Processing in Pet Nutrition. *Animals* **2025**, *16*, doi:10.3390/ani16010041.

72. Maturana, M.; Castillejos, L.; Lesaux, A.A.; Martin-Orue, S.M. Impact of an Abrupt Change from Dry to Canned Diet on Digestive Function and Gut Microbiota in Dogs. *J Anim Sci* **2025**, *103*, doi:10.1093/jas/skaf143

73. Liao, P.; Yang, K.; Huang, H.; Xin, Z.; Jian, S.; Wen, C.; He, S.; Zhang, L.; Deng, B. Abrupt Dietary Change and Gradual Dietary Transition Impact Diarrheal Symptoms, Fecal Fermentation Characteristics, Microbiota, and Metabolic Profile in Healthy Puppies. *Animals (Basel)* **2023**, *13*, doi:10.3390/ani13081300.

74. Mariti, C.; Bein, S. Evaluation of Dog Welfare before and after a Professional Grooming Session. *Dog Behavior* **2015**, *1*, doi:https://doi.org/10.4454/db.v1i1.2.

75. McDonald, S.E.; Sweeney, J.; Niestat, L.; Doherty, C. Grooming-Related Concerns among Companion Animals: Preliminary Data on an Overlooked Topic and Considerations for Animals' Access to Health-Related Services. *Frontiers in Veterinary Science* **2022**, *9*, 827348.

76. Littlewood, K.E.; Heslop, M.V.; Cobb, M.L. The Agency Domain and Behavioral Interactions: Assessing Positive Animal Welfare Using the Five Domains Model. *Frontiers in Veterinary Science* **2023**, *Volume 10-2023*, doi:10.3389/fvets.2023.1284869.

TABLE S2. Contd…

Question	Response Option	Potential Impact on Canine Welfare	Literature sources used to help assign classification
Has/will the rising cost-of-living affect your decisions regarding extra help with the day-to-day care of your dog, in a paid capacity (i.e. use of dog walkers, doggy daycare)?	Yes, I am more likely to use/have started using paid services	Potentially positive	[58,77,78]
	Yes, I am less likely to use/have stopped using paid services	Potentially negative	
	N/A – I have never needed to use paid services to help to look after my dog	None	
Given that you have used others to look after your dog when on holiday before, how has/will the rising cost-of-living affect your decision to leave your dog and/or go away on holiday?	I have switched/will switch to using a cheaper paid service to care for my dog whilst I am away	Potentially negative	[58,77]
	I have stopped using/will no longer be able to use paid services at all (e.g. boarding kennels, dog sitters) and so do/will use an unpaid service from friends/family instead	Potentially negative	
	I have gone/will now go on holidays where I can take my dog with me	Ambiguous	
	I have gone/will go on a cheaper holiday so I can afford the cost of care for my dog	Ambiguous	
	I have been/will now be unable to go on holidays at all because of the cost of care for my dog	Ambiguous	
	No change	None	
Has/will the rising cost-of-living affect the amount of time/how often your dog is left at home alone?	Yes, my dog will be left alone for less time/less often	Potentially positive	[58,78,79]
	Yes, my dog will be left alone longer/more often	Potentially negative	
	No – there will be no change in how long/often my dog is left alone as a result of rising cost-of-living	None	

57. Mellor, D.J.; Beausoleil, N.J.; Littlewood, K.E.; McLean, A.N.; McGreevy, P.D.; Jones, B.; Wilkins, C. The 2020 Five Domains Model: Including Human-Animal Interactions in Assessments of Animal Welfare. *Animals* **2020**, *10*, doi:10.3390/ani10101870.

76. Littlewood, K.E.; Heslop, M.V.; Cobb, M.L. The Agency Domain and Behavioral Interactions: Assessing Positive Animal Welfare Using the Five Domains Model. *Frontiers in Veterinary Science* **2023**, *Volume 10-2023*, doi:10.3389/fvets.2023.1284869.

77. Rehn, T.; Keeling, L.J. The Effect of Time Left Alone at Home on Dog Welfare. *Applied Animal Behaviour Science* **2011**, *129*, 129–135, doi:10.1016/j.applanim.2010.11.015.

78. Mellor, D.J. Moving beyond the "Five Freedoms" by Updating the "Five Provisions" and Introducing Aligned "Animal Welfare Aims". *Animals* **2016**, *6*, doi:10.3390/ani6100059.

TABLE S3. Demographic characteristics of UK-based owners of 36-month-old dogs purchased under 16 weeks of age between 23 March and 31 December 2020, who responded to the 'Cost-of-Living Crisis' (COLC) survey.

Category (total responses per variable)	Options	Number of responses	% of responses
**First-time dog owner in 2020 (*n* = 769)**	No	522	67.88
	Yes	247	32.12
**Owner age (*n* = 769)**	18 – 24 years	21	2.73
	25 – 34 years	100	13.00
	35 – 44 years	124	16.12
	45 – 54 years	215	27.96
	55 – 64 years	188	24.44
	65 – 74 years	108	14.04
	75 years and over	13	1.69
**Owner gender (*n* = 769)**	Male	74	9.62
	Female	695	90.38
	Other	0	0.0
	I prefer not to say	0	0.0
**Owners' current employment status (*n* = 712)**	Employed	476	66.85
	Unemployed	10	1.40
	Retired	213	29.91
	Unable to work	13	1.83
**Owners educational level (*n* = 731)**	No formal qualifications	4	0.55
	Level 1 and 2 (e.g. GCSE)	88	12.04
	Level 3 (e.g. A'Level, IB)	127	17.37
	Level 4 (e.g. CertHE, HNC)	6	0.82
	Level 5 (e.g. DipHE, HND)	5	0.68
	Level 6 (e.g. BSc, BA)	255	34.88
	Level 7 (e.g. MSc, PGCE)	205	28.04
	Level 8 (e.g. PhD)	41	5.61
**Owner lives alone/only adult in household (*n* = 752)**	No	629	83.64
	Yes	123	16.36
**Children < 18 years of age in household (*n* = 632)**	No	484	76.58
	Yes	148	23.42
**Other dog(s) currently in the household (*n* = 768)**	No	505	65.76
	Yes	263	34.24
**UK region of residence (*n* = 673)**	East Midlands	43	6.39
	East of England	98	14.56
	London	56	8.32
	North East	32	4.75
	North West	56	8.32
	Northern Ireland	3	0.45
	Scotland	60	8.92
	South East	96	14.26
	South West	82	12.18
	Wales	35	5.20
	West Midlands	58	8.62
	Yorkshire and The Humber	54	8.02

TABLE S4. Demographic characteristics of 36-month-old dogs purchased under 16 weeks of age between 23 March and 31 December 2020, whose UK-based owners responded to the 'Cost-of-Living Crisis' (COLC) survey.

Category (total responses per variable)	Options	Number of responses	% of total responses per variable
**Dog sex (*n* = 769)**	Male	395	51.37
	Female	374	48.63
**Neuter status (*n* = 751)**	Neutered	551	73.37
	Entire	200	26.63
**Breed/crossbreed categorisation (*n* = 769)**	Crossbreed	29	3.77
	Designer Crossbreed	190	24.71
	Purebreed	550	71.52
**Poodle-type categorisation (*n* = 740)**	Non-Poodle-type	591	79.86
	Poodle-type purebred	9	1.22
	Poodle-type crossbreed	140	18.92
**Top 12 most common breeds at 36 months (*n* = 774)**	Labrador Retriever	80	10.34
	Cockapoo	59	7.62
	Cocker Spaniel	48	6.20
	Border Collie	37	4.78
	Crossbreed	29	3.75
	Border Terrier	26	3.36
	Golden Retriever	24	3.10
	Miniature Smooth-Haired Dachshund	23	2.97
	Cavapoo	18	2.33
	Labradoodle	18	2.33
	Sprocker	16	2.07
	English Springer Spaniel	15	1.94
	Other	381	49.22
**UK Royal Kennel Club (RKC) breed group (*n* = 769)**	Not RKC recognised	226	29.39
	Gundog	230	29.91
	Hound	63	8.19
	Pastoral	69	8.97
	Terrier	66	8.58
	Toy	30	3.90
	Utility	51	6.63
	Working	34	4.42
**Dogs' mean adult bodyweight categorisation (*n* = 747)**	≤ 10 kg	162	21.69
	10 to < 20 kg	282	37.75
	20 to < 30 kg	171	22.89
	30 to < 40 kg	111	14.86
	≥ 40 kg	21	2.81

TABLE S5. Descriptive and univariable binary logistic regression analysis of changes in **decisions regarding insurance cover and preventative healthcare provision** for their dog, as a result of the Cost-of-Living Crisis (COLC), amongst UK-based owners of 36-month-old dogs purchased in the UK under 16 weeks of age between 23 March and 31 December 2020. Changes were categorised as a binary outcome of no change/positive change versus changes with the potential for negative canine welfare. Significant differences (*P* < 0.050) are emboldened. *95% Confidence Interval. uOR unadjusted odds ratio.

Health care provision	Variable (*n* = total responses per variable)	Category	*n* no change/positive change vs. negative change	% negative change	uOR	95% CI*	*P*-Value
Insurance Cover	Dog ownership experience in 2020 (*n* = 765)	First-time owner	207 vs. 38	15.51	0.889	0.587 – 1.375	0.578
		Previous dog owner	431 vs. 89	17.12		Ref	
	Owners' age in 2020 (*n* = 765)	**18 – 24 years**	**12 vs. 9**	**42.86**	**4.452**	**1.731 – 11.445**	**0.002**
		25 – 34 years	81 vs. 17	17.35	1.246	0.652 – 2.378	0.505
		**35 – 44 years**	**116 vs. 8**	**6.45**	**0.409**	**0.182 – 0.921**	**0.031**
		45 – 54 years	184 vs. 31	14.42		Ref	
		55 – 64 years	154 vs. 33	17.65	1.272	0.745 – 2.172	0.378
		**65 – 74 years**	**82 vs. 25**	**23.36**	**1.810**	**1.005 – 3.257**	**0.048**
		75 years and over	9 vs. 4	30.77	2.638	0.765 – 9.095	0.125
	Current annual household income (*n* = 651)	≤ £30,000	**87 vs. 26**	**23.01**	**3.415**	**1.696 – 6.879**	**< 0.001**
		£30,001 – £60,000	**169 vs. 48**	**22.12**	**3.246**	**1.723 – 6.115**	**< 0.001**
		£60,001 – £90,000	125 vs. 22	14.97	2.011	0.989 – 4.090	0.054
		≥ £90,001	160 vs. 14	8.05		Ref	
	Current employment status (*n* = 709)	Employed	408 vs. 67	14.11		Ref	
		Unemployed	9 vs. 1	10.00	0.677	0.084 – 5.427	0.713
		**Retired**	**166 vs. 45**	**21.33**	**1.651**	**1.086 – 2.509**	**0.019**
		Unable to work	10 vs. 3	23.08	1.827	0.490 – 6.810	0.369
	Dogs' mean adult bodyweight (*n* = 739)	≤ 10 kg	131 vs. 29	18.13	1.182	0.706 – 1.979	0.524
		10 to < 20 kg	235 vs. 44	15.77		Ref	
		20 to < 30 kg	145 vs. 25	14.71	0.921	0.541 – 1.569	0.762
		30 to < 40 kg	92 vs. 17	15.60	0.987	0.537 – 1.815	0.966
		**≥ 40 kg**	**14 vs. 7**	**33.33**	**2.670**	**1.020 – 6.993**	**0.046**
	Dog attended veterinary clinic for health issue in previous 9 months (*n* = 744)	No	353 vs. 72	16.94		Ref	
		Yes	268 vs. 51	15.99	0.933	0.630 – 1.381	0.729
	Dog has ongoing health issues requiring veterinary visits (*n* = 303)	No	186 vs. 41	18.06		Ref	
		**Yes**	**71 vs. 5**	**6.58**	**0.319**	**0.121 – 0.841**	**0.021**

TABLE S5. **Contd…**

Health care provision	Variable (*n* = total responses per variable)	Category	*n* no change/positive change vs. negative change	% negative change	uOR	95% CI*	*P*-Value
Worm treatment	Dog ownership experience in 2020 (*n* = 701)	First-time owner	218 vs. 11	4.80	0.832	0.405 – 1.708	0.616
		Previous dog owner	445 vs. 27	5.72		Ref	
	Owners' age in 2020 (*n* = 701)	**18 – 24 years**	**13 vs. 4**	**23.53**	**5.315**	**1.458 – 19.017**	**0.010**
		25 – 34 years	86 vs. 5	5.49	1.004	0.339 – 2.979	0.994
		35 – 44 years	109 vs. 7	6.03	1.109	0.418 – 2.945	0.835
		45 – 54 years	190 vs. 11	5.47		Ref	
		55 – 64 years	165 vs. 5	2.94	0.523	0.178 – 1.537	0.239
		65 – 74 years	90 vs. 4	4.26	0.768	0.238 – 2.477	0.658
		75 years and over	10 vs. 2	16.67	3.455	0.673 – 17.726	0.137
	Current annual household income (*n* = 598)	≤ £30,000	88 vs. 7	7.37	2.561	0.790 – 8.308	0.117
		£30,001 – £60,000	190 vs. 13	6.40	2.203	0.769 – 6.312	0.141
		£60,001 – £90,000	126 vs. 8	5.97	2.044	0.653 – 6.402	0.219
		≥ £90,001	161 vs. 5	3.01		Ref	
	Current employment status (*n* = 649)	Employed	141 vs. 23	5.26		Ref	
		Unemployed	7 vs. 1	12.50	2.571	0.303 – 21.789	0.386
		Retired	180 vs. 11	5.76	1.100	0.525 – 2.304	0.801
		Unable to work	12 vs. 1	7.69	1.500	0.187 – 12.040	0.703
	Dogs' mean adult bodyweight (*n* = 675)	≤ 10 kg	137 vs. 11	7.43	1.781	0.753 – 4.215	0.189
		10 to < 20 kg	244 vs. 11	4.31		Ref	
		20 to < 30 kg	142 vs. 8	5.33	1.250	0.491 – 3.180	0.640
		30 to < 40 kg	98 vs. 4	3.92	0.950	0.282 – 2.912	0.868
		≥ 40 kg	17 vs. 3	15.00	3.914	0.997 – 15.374	0.051
	Dog attended veterinary clinic for health issue in previous 9 months (*n* = 683)	No	367 vs. 25	6.38		Ref	
		Yes	279 vs. 12	4.12	0.631	0.312 – 1.279	0.202
	Dog has ongoing health issues requiring veterinary visits (*n* = 277)	No	199 vs. 6	2.93		Ref	
		Yes	67 vs. 5	6.94	2.475	0.732 – 8.373	0.145

TABLE S5. **Contd…**

Health care provision	Variable (*n* = total responses per variable)	Category	*n* no change/positive change vs. negative change	% negative change	uOR	95% CI*	*P*-Value
Flea treatment	Dog ownership experience in 2020 (*n* = 704)	First-time owner	216 vs. 16	6.90	1.221	0.644 – 2.314	0.541
		Previous dog owner	445 vs. 27	5.72		Ref	
	Owners' age in 2020 (*n* = 704)	**18 – 24 years**	**13 vs. 5**	**27.78**	**12.564**	**3.380 – 46.699**	**< 0.001**
		**25 – 34 years**	**84 vs. 8**	**8.70**	**3.111**	**1.047 – 9.243**	**0.041**
		35 – 44 years	196 vs. 6	2.97	2.398	0.811 – 7.089	0.114
		45 – 54 years	109 vs. 8	6.84		Ref	
		55 – 64 years	163 vs. 8	4.68	1.603	0.545 – 4.715	0.391
		65 –74 years	87 vs. 5	5.43	1.877	0.558 – 6.317	0.309
		**75 years and over**	**9 vs. 3**	**25.00**	**10.889**	**2.338 – 50.719**	**0.002**
	Current annual household income (*n* = 599)	**≤ £30,000**	**85 vs. 9**	**9.57**	**3.388**	**1.101 – 10.430**	**0.033**
		£30,001 – £60,000	186 vs. 13	6.53	2.237	0.780 – 6.409	0.134
		£60,001 – £90,000	132 vs. 9	6.38	2.182	0.714 – 6.669	0.171
		≥ £90,001	160 vs. 5	3.03		Ref	
	Current employment status (*n* = 652)	Employed	416 vs. 25	5.67		Ref	
		Unemployed	7 vs. 1	12.50	2.377	0.281 – 20.080	0.426
		Retired	178 vs. 12	6.32	1.122	0.551 – 2.283	0.751
		Unable to work	12 vs. 1	7.69	1.387	0.173 – 11.094	0.758
	Dogs' mean adult bodyweight (*n* = 680)	≤ 10 kg	134 vs. 11	7.59	1.046	0.483 – 2.263	0.910
		10 to < 20 kg	242 vs. 19	7.28		Ref	
		20 to < 30 kg	146 vs. 6	3.95	0.523	0.204 – 1.341	0.177
		30 to < 40 kg	97 vs. 5	4.90	0.657	0.238 – 1.808	0.416
		≥ 40 kg	18 vs. 2	10.00	1.415	0.305 – 6.560	0.657
	Dog attended veterinary clinic for health issue in previous 9 months (*n* = 685)	No	369 vs. 28	7.05		Ref	
		Yes	274 vs. 14	4.86	0.673	0.348 – 1.303	0.240
	Dog has ongoing health issues requiring veterinary visits (*n* = 273)	No	191 vs. 10	4.98		Ref	
		Yes	69 vs. 3	4.17	0.830	0.222 – 3.106	0.783

TABLE S6. Descriptive and univariable binary logistic analysis of changes in respondents **decisions regarding seeking advice from a veterinary surgeon for their dogs health problems in the preceding 9 months**, as a result of the Cost-of-Living Crisis (COLC), amongst UK-based owners of 36-month-old dogs purchased in the UK under 16 weeks of age between 23 March and 31 December 2020. Changes were categorised as a binary outcome of no change/ /positive change versus changes with the potential for negative canine welfare. Significant differences (*P* < 0.050) are emboldened. ~ No analysis possible. *95% Confidence Interval. IQR Interquartile Range. uOR unadjusted odds ratio.

	**Variable (*n* = total responses per variable)**	**Category**	***n* no change/positive change vs. negative change**	**% negative change**	**uOR**	**95% CI***	***P*-Value**
**Owner/ household demographics**	Dog ownership experience in 2020 (*n* = 482)	First-time owner	138 vs. 27	16.36	1.281	0.758 – 2.165	0.355
		Previous dog owner	275 vs. 42	13.25		Ref	
	Owners' age in 2020 (*n* = 482)	**18 – 24 years**	**6 vs. 5**	**45.45**	**4.683**	**1.309 – 16.747**	**0.018**
		25 – 34 years	55 vs. 12	17.91	1.226	0.536 – 2.669	0.608
		35 – 44 years	79 vs. 15	15.96	1.067	0.519 – 2.195	0.860
		45 – 54 years	118 vs. 21	15.11		Ref	
		55 – 64 years	96 vs. 11	10.28	0.644	0.296 – 1.401	0.267
		65 – 74 years	53 vs. 5	8.62	0.530	0.190 – 1.481	0.226
		75 years and over	6 vs. 0	0.00	~	~	~
	Current annual household income (*n* = 413)	≤ £30,000	48 vs. 9	15.79	1.766	0.698 – 4.466	0.230
		£30,001 – £60,000	103 vs. 22	17.60	2.011	0.948 – 4.268	0.069
		£60,001 – £90,000	90 vs. 16	15.09	1.674	0.754 – 3.718	0.206
		≥ £90,001	113 vs. 12	9.60		Ref	
	Current employment status (*n* = 445)	Employed	256 vs. 50	16.34		Ref	
		Unemployed	7 vs. 2	22.22	1.463	0.295 – 7.249	0.641
		Retired	111 vs. 12	9.76	0.554	0.284 – 1.080	0.083
		Unable to work	7 vs. 0	0.00	~	~	~

TABLE S6. Contd…

	Variable (*n* = total responses per variable)	Category	*n* no change/positive change vs. negative change	% negative change	uOR	95% CI*	*P*-Value
**Dog demographics**	Dogs' mean adult bodyweight (*n* = 468)	≤ 10 kg	89 vs. 17	16.04	1.036	0.532 – 2.017	0.917
		10 to < 20 kg	141 vs. 26	15.57		Ref	
		20 to < 30 kg	86 vs. 14	14.00	0.883	0.437 – 1.783	0.728
		30 to < 40 kg	70 vs. 8	10.26	0.620	0.267 – 1.439	0.266
		≥ 40 kg	15 vs. 2	11.76	0.723	0.156 – 3.351	0.679
**Purchase motivations for breed/ crossbreed**	Affordable cost of upkeep (*n* = 483)	No	387 vs. 58	13.03		Ref	
		**Yes**	**27 vs. 11**	**28.95**	**2.718**	**1.280 – 5.775**	**0.009**
	Generally healthy (*n* = 483)	No	198 vs. 37	15.74		Ref	
		Yes	216 vs. 32	12.90	0.476 – 1.321	0.793	0.373
**Owner expectations**	Veterinary costs (*n* = 458)	Less than/as expected	304 vs. 42	12.14		Ref	
		**More than expected**	**90 vs. 22**	**19.67**	**1.769**	**1.004 – 3.119**	**0.049**
**Dog health**	Attended veterinary clinic for health issue in previous 9 months (*n* = 467)	No	166 vs. 18	9.78		Ref	
		**Yes**	**235 vs. 48**	**16.96**	**1.884**	**1.058 – 3.354**	**0.031**
	Number of visits to veterinary clinic for health problem in preceding 9 months (*n* = 482)	/ (Median = 2.00, Range = 1 – 20, IQR = 1.00 – 3.00)	234 vs. 48	17.02	1.015	0.897 – 1.148	0.816
	Number of health disorder groups requiring visits to veterinary clinic in preceding 9 months (*n* = 483)	/ (Median = 0.00, Range = 0 – 5, IQR = 0.00 – 1.00)	414 vs. 69	14.29	1.268	0.926 – 1.736	0.139
	Ongoing health issues requiring veterinary visits (*n* = 267)	No	163 vs. 31	15.98		Ref	
		Yes	59 vs. 14	19.18	1.248	0.621 – 2.507	0.534
	Total spend on health disorders at veterinary clinic(s) in preceding 9 months (*n* = 259)	/ (Median = £250.00, Range = £13.00 – £9200.00, IQR = £100.00 – £700.00)	213 vs. 46	17.76	1.000	1.000 – 1.000	0.714
	**Number of health disorder groups in the preceding 9 months, whether or not veterinary advice sought (*n* = 400)**	**/** (Median = 1.00, Range = 0.00 – 8.00, IQR = 1.00 – 2.00)	**344 vs. 56**	**14.00**	**1.397**	**1.169 – 1.669**	**< 0.001**

TABLE S6. Contd…

	Variable (*n* = total responses per variable)	Category	*n* no change/positive change vs. negative change	% negative change	uOR	95% CI*	*P*-Value
**Dog health**	Vaccinations up to date (*n* = 441)	**No**	**29 vs. 11**	**27.50**	**2.546**	**1.199 – 5.404**	**0.015**
		Yes	349 vs. 52	12.97		Ref	
	Insurance status (*n* = 461)	Insured	349 vs. 52	12.97		Ref	
		**Not insured**	**46 vs. 14**	**23.33**	**2.043**	**1.050 – 3.974**	**0.035**
**COLC impacts, dog health**	Change in decision to insure dog (*n* = 480)	No/positive change	359 vs. 52	12.65		Ref	
		**Negative change**	**52 vs. 17**	**24.64**	**2.257**	**1.214 – 4.196**	**0.010**
	Change in decision to use worming treatments (*n* = 448)	No/positive change	383 vs. 46	10.72		Ref	
		**Negative change**	**10 vs. 9**	**47.37**	**7.493**	**2.895 – 19.397**	**< 0.001**
	Change in decision to use flea treatments (*n* = 453)	No/positive change	387 vs. 44	10.21		Ref	
		**Negative change**	**8 vs. 14**	**63.64**	**15.392**	**6.116 – 38.737**	**< 0.001**
**COLC impacts, dog maintenance**	Change in dogs' diet in the preceding 9 months (*n* = 480)	No/non-COLC change	369 vs. 45	10.87		Ref	
		**COLC change**	**44 vs. 22**	**33.33**	**4.100**	**2.254 – 7.456**	**< 0.001**
**COLC impacts, household finances and negative canine welfare**	Change in household finances in preceding 9 months (*n* = 467)	**Worse**	**144 vs. 42**	**22.58**	**3.208**	**1.793 – 5.742**	**< 0.001**
		Better	48 vs. 5	9.43	1.146	0.407 – 3.222	0.796
		No change	209 vs. 19	8.33		Ref	
	Change in household circumstances in next 6 months (*n* = 449)	**Harder to keep my dog**	**34 vs. 17**	**33.33**	**4.742**	**2.393 – 9.397**	**< 0.001**
		Easier to keep my dog	44 vs. 8	15.38	1.725	0.749 – 3.972	0.201
		No change	313 vs. 33	9.54		Ref	
	Negative impact on dog care currently (*n* = 476)	No	372 vs. 41	9.93		Ref	
		**Yes**	**38 vs. 25**	**39.68**	**5.969**	**3.279 – 10.865**	**< 0.001**
	Negative impact on dog care in the next 6 months (*n* = 448)	No	351 vs. 35	9.07		Ref	
		**Yes**	**36 vs. 26**	**41.94**	**7.243**	**3.926 – 13.363**	**< 0.001**
	Owning a dog is causing a negative impact on household lifestyle currently (*n* = 468)	No	278 vs. 18	6.08		Ref	
		**Yes**	**128 vs. 44**	**25.58**	**5.309**	**2.952 – 9.549**	**< 0.001**
	Owning a dog will cause a negative impact on household lifestyle in the next 6 months (*n* = 450)	No	279 vs. 21	7.00		Ref	
		**Yes**	**110 vs. 40**	**26.67**	**4.831**	**2.726 – 8.563**	**< 0.001**

TABLE S7. Descriptive and univariable binary logistic analysis of changes in respondents **decisions regarding seeking advice for their dogs behavioural problems in the preceding 9 months**, as a result of the Cost-of-Living Crisis (COLC), amongst UK-based of 36-month-old dogs purchased in the UK under 16 weeks of age between 23 March and 31 December 2020. Changes were categorised as a binary outcome of no change/positive change versus those with the potential for negative canine welfare. Significant differences ($P < 0.050$) are emboldened. ~ No analysis possible. *95% Confidence Interval. IQR Interquartile Range. uOR unadjusted odds ratio.

	Variable (*n* = total responses per variable)	Category	*n* no change/positive change vs. negative change	% negative change	uOR	95% CI*	*P*-Value
**Owner/ household demographics**	Dog ownership experience in 2020 (*n* = 258)	No	65 vs. 21	24.42	0.940	0.516 – 1.712	0.839
		Yes	128 vs. 44	25.58		Ref	
	Owners' age in 2020 (*n* = 258)	**18 – 24 years**	**1 vs. 4**	**80.00**	**11.455**	**1.214 – 108.073**	**0.033**
		25 – 34 years	26 vs. 8	23.53	0.881	0.348 – 2.232	0.790
		35 – 44 years	38 vs. 8	17.39	0.603	0.244 – 1.488	0.272
		45 – 54 years	63 vs. 22	25.88		Ref	
		55 – 64 years	40 vs. 13	35.85	0.931	0.422 – 2.055	0.859
		65 – 74 years	24 vs. 6	20.00	0.716	0.259 – 1.981	0.520
		75 years and over	2 vs. 3	60.00	4.295	0.673 – 27.424	0.123
	Current annual household income (*n* = 215)	**≤ £30,000**	**27 vs. 13**	**32.50**	**2.829**	**1.041 – 7.688**	**0.042**
		**£30,001 – £60,000**	**39 vs. 20**	**33.90**	**3.013**	**1.197 – 7.585**	**0.019**
		£60,001 – £90,000	44 vs. 17	27.87	2.270	0.891 – 5.785	0.086
		≥ £90,001	47 vs. 8	14.55		Ref	
	Current employment status (*n* = 239)	Employed	123 vs. 41	25.00		Ref	
		Unemployed	1 vs. 1	50.00	3.000	0.183 – 49.051	0.441
		Retired	53 vs. 17	24.29	0.962	0.502 – 1.844	0.908
		Unable to work	2 vs. 1	33.33	1.500	0.133 – 16.976	0.743
**Dog demographics**	Dogs' mean adult bodyweight (*n* = 250)	≤ 10 kg	39 vs. 18	31.58	1.442	0.700 – 2.973	0.321
		10 to < 20 kg	75 vs. 24	24.24		Ref	
		20 to < 30 kg	37 vs. 10	21.28	0.845	0.366 – 1.949	0.692
		30 to < 40 kg	31 vs. 8	20.51	0.806	0.327 – 1.989	0.641
		≥ 40 kg	6 vs. 2	25.00	1.042	0.197 – 5.506	0.962

TABLE S7. Contd…

	Variable (*n* = total responses per variable)	Category	*n* no change/positive change vs. negative change	% negative change	uOR	95% CI*	*P*-Value
**Purchase motivations for breed/ crossbreed**	Easy to train (*n* = 259)	No	107 vs. 42	28.19		Ref	
		Yes	87 vs. 23	20.91	0.674	0.376 – 1.205	0.183
**Owner expectations**	Training (*n* = 245)	Easier than/as expected	124 vs. 32	20.51		Ref	
		**Harder than expected**	**60 vs. 29**	**32.58**	**1.873**	**1.039 – 3.377**	**0.037**
	Behaviour (*n* = 239)	Better than/as expected	142 vs. 40	21.98			
		Worse than expected	38 vs. 19	33.33	1.775	0.924 – 3.410	0.085
**Dog behaviour**	Displayed a 'control behaviour' in the preceding 9 months (*n* = 236)	No	44 vs. 3	6.38		Ref	
		**Yes**	**134 vs. 55**	**29.10**	**6.020**	**1.793 – 20.206**	**0.004**
	Displayed an 'attention-seeking behaviour' in the preceding 9 months (*n* = 197)	No	54 vs. 15	21.74		Ref	
		Yes	91 vs. 37	28.91	1.464	0.736 – 2.912	0.278
	Displayed an 'aggressive behaviour' in the preceding 9 months (*n* = 237)	No	112 vs. 27	19.42		Ref	
		**Yes**	**67 vs. 31**	**31.63**	**1.919**	**1.055 – 3.491**	**0.033**
	Displayed a 'fear/avoidance behaviour' in the preceding 9 months (*n* = 237)	No	85 vs. 15	15.00		Ref	
		**Yes**	**94 vs. 43**	**31.39**	**2.592**	**1.344 – 5.000**	**0.004**
	Displayed a 'reaction to familiar people behaviour' in the preceding 9 months (*n* = 238)	No	171 vs. 49	22.27		Ref	
		**Yes**	**9 vs. 9**	**50.00**	**3.490**	**1.314 – 9.271**	**0.012**
	Displayed a 'reaction to unfamiliar people behaviour' in the preceding 9 months (*n* = 239)	No	128 vs. 35	21.47		Ref	
		Yes	53 vs. 23	30.26	1.587	0.857 – 2.938	0.142
	Displayed a 'reaction to dogs' behaviour' in the preceding 9 months (*n* = 240)	No	106 vs. 30	22.06		Ref	
		Yes	74 vs. 30	28.85	1.432	0.797 – 2.576	0.230

TABLE S7. Contd…

	**Variable (*n* = total responses per variable)**	**Category**	***n* no change/positive change vs. negative change**	**% negative change**	**uOR**	**95% CI***	***P*-Value**
**Dog behaviour**	**Number of 'problem behaviours' in the preceding 9 months (*n* = 187)**	**/** (Median = 3.00, Range = 1 – 15, IQR = 2.00 – 5.00)	**137 vs. 50**	**26.74**	**1.250**	**1.100 – 1.419**	**< 0.001**
**COLC impacts, dog training**	Change in decision regarding dog training (*n* = 169)	No change	112 vs. 14	11.11		Ref	
		**Change**	**12 vs. 31**	**72.09**	**20.667**	**8.679 – 49.211**	**< 0.001**
**COLC impacts, household finances and negative canine welfare**	Change in household finances in preceding 9 months (*n* = 264)	**Worse**	**68 vs. 39**	**36.45**	**2.294**	**1.255 – 4.193**	**0.007**
		Better	22 vs. 2	8.33	0.364	0.080 – 1.659	0.191
		No change	92 vs. 23	20.00		Ref	
	Change in household circumstances in next 6 months (*n* = 238)	**Harder to keep my dog**	**22 vs. 14**	**38.89**	**2.459**	**1.149 – 5.266**	**0.021**
		Easier to keep my dog	16 vs. 6	27.28	1.449	0.530 – 3.961	0.469
		No change	143 vs. 37	20.56		Ref	
	Negative impact on dog care currently (*n* = 255)	No	171 vs. 39	18.57		Ref	
		**Yes**	**20 vs. 25**	**55.56**	**5.481**	**2.768 – 10.853**	**< 0.001**
	Negative impact on dog care in the next 6 months (*n* = 235)	No	161 vs. 36	18.27		Ref	
		**Yes**	**19 vs. 19**	**50.00**	**4.472**	**2.152 – 9.293**	**< 0.001**
	Owning a dog is causing a negative impact on household lifestyle currently (*n* = 247)	No	118 vs. 26	18.06		Ref	
		**Yes**	**68 vs. 35**	**33.98**	**2.336**	**1.297 – 4.209**	**0.005**
	Owning a dog will cause a negative impact on household lifestyle in the next 6 months (*n* = 236)	No	118 vs. 25	17.48		Ref	
		**Yes**	**58 vs. 35**	**37.63**	**2.848**	**1.560 – 5.199**	**< 0.001**
